# A 1.7‐Mb chromosomal inversion downstream of a *PpOFP1* gene is responsible for flat fruit shape in peach

**DOI:** 10.1111/pbi.13455

**Published:** 2020-08-17

**Authors:** Hui Zhou, Ruijuan Ma, Lei Gao, Jinyun Zhang, Aidi Zhang, Xiujun Zhang, Fei Ren, Weihan Zhang, Liao Liao, Qiurui Yang, Shengli Xu, Collins Otieno Ogutu, Jianbo Zhao, Mingliang Yu, Quan Jiang, Schuyler S. Korban, Yuepeng Han

**Affiliations:** ^1^ CAS Key Laboratory of Plant Germplasm Enhancement and Specialty Agriculture Wuhan Botanical Garden The Innovative Academy of Seed Design Chinese Academy of Sciences Wuhan China; ^2^ Key Laboratory of Genetic Improvement and Ecophysiology of Horticultural Crops Institute of Horticulture Anhui Academy of Agricultural Sciences Hefei China; ^3^ Center of Economic Botany Core Botanical Gardens Chinese Academy of Sciences Wuhan China; ^4^ Institute of Horticulture Jiangsu Academy of Agricultural Sciences Nanjing China; ^5^ Boyce Thompson Institute for Plant Research Cornell University Ithaca NY USA; ^6^ Institute of Forestry and Pomology Beijing Academy of Agriculture and Forestry Sciences Beijing China; ^7^ Agricultural Bioinformatics Key Laboratory of Hubei Province College of Informatics Huazhong Agricultural University Wuhan China; ^8^ Department of Natural Resources and Environmental Sciences University of Illinois at Urbana‐Champaign Urbana IL USA; ^9^ Sino‐African Joint Research Center Chinese Academy of Sciences Wuhan China

**Keywords:** *Prunus*, flat peach, chromosomal inversion, ovate family protein, TONNEAU1‐recruiting motif protein

## Abstract

Flat peaches have become popular worldwide due to their novelty and convenience. The peach flat fruit trait is genetically controlled by a single gene at the *S* locus, but its genetic basis remains unclear. Here, we report a 1.7‐Mb chromosomal inversion downstream of a candidate gene encoding OVATE Family Protein, designated *PpOFP1*, as the causal mutation for the peach flat fruit trait. Genotyping of 727 peach cultivars revealed an occurrence of this large inversion in flat peaches, but absent in round peaches. Ectopic overexpression of *PpOFP1* resulted in oval‐shaped leaves and shortened siliques in *Arabidopsis*, suggesting its role in repressing cell elongation. Transcriptional activation of *PpOFP1* by the chromosomal inversion may repress vertical elongation in flat‐shaped fruits at early stages of development, resulting in the flat fruit shape. Moreover, *PpOFP1* can interact with fruit elongation activator *PpTRM17*, suggesting a regulatory network controlling fruit shape in peach. Additionally, screening of peach wild relatives revealed an exclusive presence of the chromosomal inversion in *P. ferganensis*, supporting that this species is the ancestor of the domesticated peach. This study provides new insights into mechanisms underlying fruit shape evolution and molecular tools for genetic improvement of fruit shape trait in peach breeding programmes.

## Introduction

Peach (*Prunus persica* L. Batsch) is one of the most economically important fruit trees worldwide. Peach originated about 2.5 Mya in the southwest range of the Tibetan Plateau in China (Yu *et al*., 2018), and its cultivation and domestication in China can be traced back to at least 4000 years ago (Scorza and Okie, 1991; Wang, 1985). Peach has a wide range of variation for exterior fruit quality traits, with lack of skin pubescence (nectarine) and flat fruit shape (flat peach or donut peach) being the most remarkable and distinguishable (Vendramin *et al*., 2014). The flat fruit shape was initially not considered as an important trait due to its effects on fruit size, yield and fruit cracking (Dirlewanger *et al*., 1999). Thus, the flat fruit shape trait was first negatively selected in most breeding programs in western countries (Picañol *et al*., 2013). However, flat peaches have recently attracted more interest because of their high quality fruits with relatively low acidity and high sugar content (Ma *et al*., 2003). Flat peaches originated in China and have become more popular in Chinese markets because of their native name ‘Pantao’, which symbolizes health and immortality as mentioned in a famous 16th Century Chinese mythology novel ‘Journey to the West’. The flat peach is a natural mutation of the round peach and was introduced to Western countries from China in 17th Century (Faust and Timon, 1995).

Peach is a diploid species, with a small genome of ~265 Mb (Verde *et al*., 2013). The flat fruit trait is genetically controlled by a single *S* locus (Lesley, 1940), which was mapped to the bottom of chromosome (Chr) 6 (Dirlewanger *et al*., 1998). The *S* locus is within a chromosomal interval that harbours quantitative trait loci (QTLs) for fresh weight and productivity, which explains why flat peaches are less productive and bear light‐weight fruits (Dirlewanger *et al*., 1999). In addition, the *S* locus is also tightly linked to a major QTL controlling the phenomenon of fruit abortion in segregating F_2_ progenies (Dirlewanger *et al*., 2006), but it remains unclear whether fruit abortion and the flat fruit trait are determined by the same gene at the *S* locus.

To conduct marker‐assisted selection (MAS) in breeding programs, simple sequence repeat (SSR) markers linked to the flat fruit trait were developed (Dirlewanger *et al*., 2004; Howad *et al*., 2005), with one SSR UDP98‐412 having a 98.4% accuracy for predicting the flat shape phenotype (Picañol *et al*., 2013). A genome‐wide association study (GWAS) of 129 peach accessions revealed a candidate gene *CAD1* (constitutively activated cell death 1, ppa003772m) for the peach flat fruit trait as an A‐T single nucleotide polymorphism (SNP) within its intron co‐segregates with the flat fruit trait (Cao *et al*., 2016). An additional study demonstrated that a ∼10 Kb deletion of the promoter and partial coding regions of a candidate gene *LRR‐RLK* encoding leucine‐rich repeat receptor‐like kinase (PRUPE.6G281100) co‐segregates with the flat fruit trait in a collection of 246 cultivars (López‐Girona *et al*., 2017). The *CAD1* and *LRR‐RLK* genes are separated by an interval of over 600 Kb in length, which overlaps a large linkage disequilibrium (LD) block on Chr6 (Aranzana *et al*., 2010; Cao *et al*., 2014), resulting in their tight linkage with the flat fruit trait. However, a recent study indicates that the *CAD1* gene is unlikely the causal gene for the peach flat fruit trait, and the ∼10 Kb deletion associated with the *LRR‐RLK* gene fails to co‐segregate with the flat fruit trait in peach germplasm (Guo *et al*., 2018). Hence, more studies are still needed to elucidate the genetic basis of the flat fruit trait in peach.

Tomato has abundant fruit shape variations, which provide opportunities to comprehensively investigate QTLs controlling fruit shape (Brewer *et al*., 2007; Gonzalo and van der Knaap, 2008; Rodriguez *et al*., 2011). To date, four candidate genes controlling fruit shape, *SUN*, *LOCULE NUMBER (LC)*, *FASCIATED (FAS)* and *OVATE*, which encode an IQ67 domain protein, the WUSCHEL homeodomain protein, a YABBY transcription factor and an OVATE family protein (OFP), respectively, have been identified in tomato. Both *LC* and *FAS* control locule number, thus, influencing fruit shape (Cong *et al*., 2008; Muños *et al*., 2011), whereas, *SUN* and *OVATE* are both involved in the regulation of fruit elongation. *SUN* is a positive regulator of fruit elongation (Wu *et al*., 2011), while *OVATE* is a repressive regulator of growth resulting in shorter fruit (Liu *et al*., 2002; Wu *et al*., 2018). *OVATE* is the first member of the OFP family identified in plants, which share a conserved 70 amino acid OVATE domain, also known as DUF623 domain, in the C‐terminus (Liu *et al*., 2014). A recent study reveals that the elongated fruit phenotype in tomato is caused by a simultaneous null mutation of *OVATE* and a deletion in the upstream regulatory region of another *OVATE‐like* gene *SlOFP20* (Wu *et al*., 2018). *OFPs* in *Arabidopsis* and cotton were also proved to be transcriptional repressors in cell division or elongation (Wang *et al*., 2016; Wang *et al*., 2007; Yang *et al*., 2018). In addition, the *OFP* genes can interact with other TFs such as encoding TONNEAU1‐recruiting motif proteins to repress cell division in ovary development (Li *et al*., 2011; Wu *et al*., 2018).

In this study, we report a 1.7‐Mb chromosomal inversion located ~3 Kb downstream of the stop codon of a candidate gene for the peach flat fruit trait, designated *PpOFP1*, a member of OFP family. This inversion event was detected in peach accessions with flat‐shaped fruits, but not in those with round‐shaped fruits. The *PpOFP1* expression was activated by the 1.7‐Mb chromosomal inversion in flat‐shaped fruits at early stages of development, resulting in flat‐shaped fruit. Our study offers new insights into molecular mechanisms underlying the flat fruit trait in peach and provides molecular tools for genetic improvement of fruit shape in peach breeding programs.

## Results

### Identification of a 1.7‐Mb chromosomal inversion in the *S* locus in peach using PacBio sequencing

**Figure 1:**
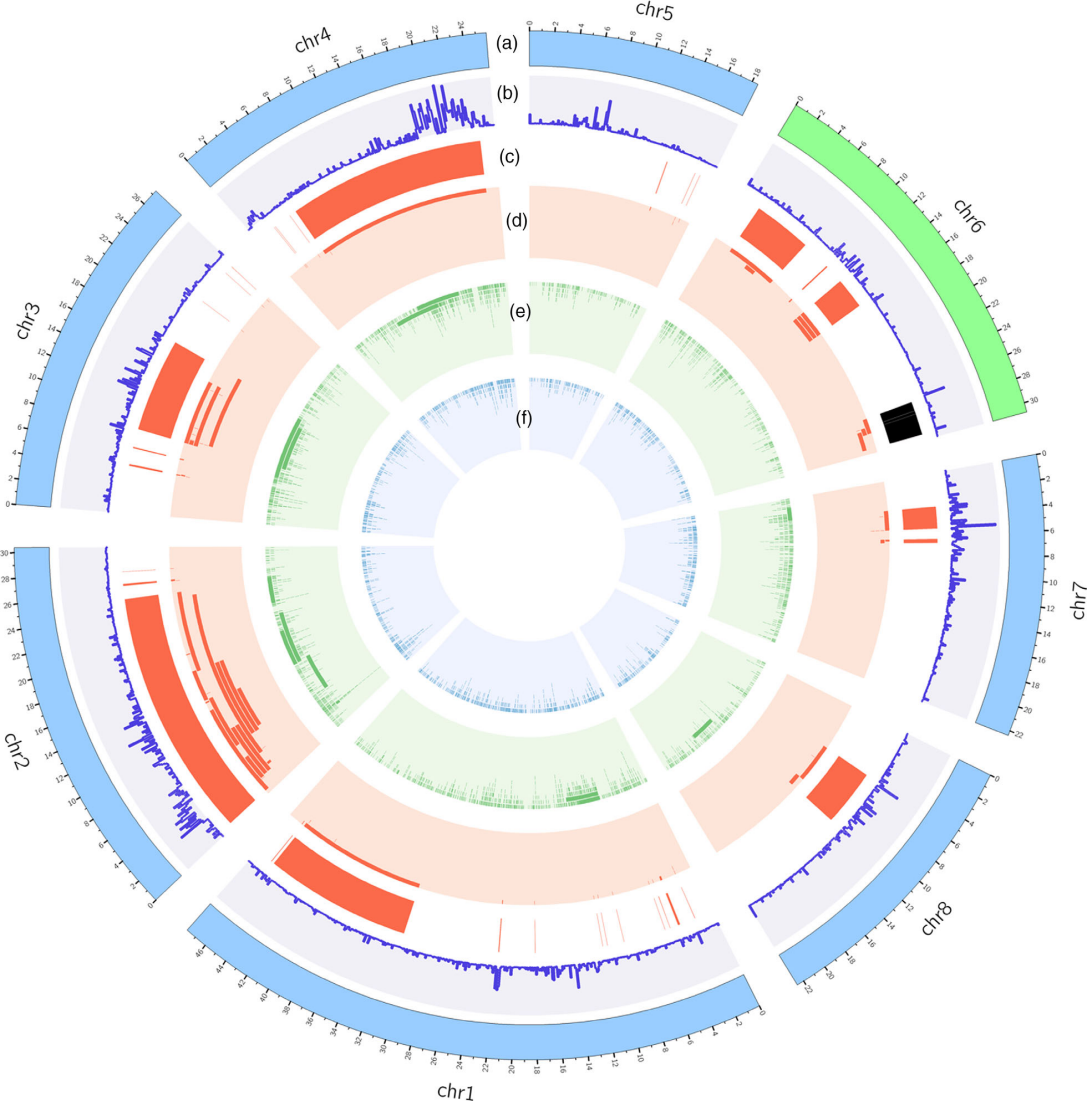
Circos plot shows overall distribution SVs in the genome. Tracks from outside to inside are chromosomes ideograms (a), SNP density (b), highlights of inversions (c), and tile plots of inversions (d), deletions (e) and insertions (f). The 1.7‐Mb inversion located at the S locus on chr6 is highlighted in black colour.

**Figure 2:**
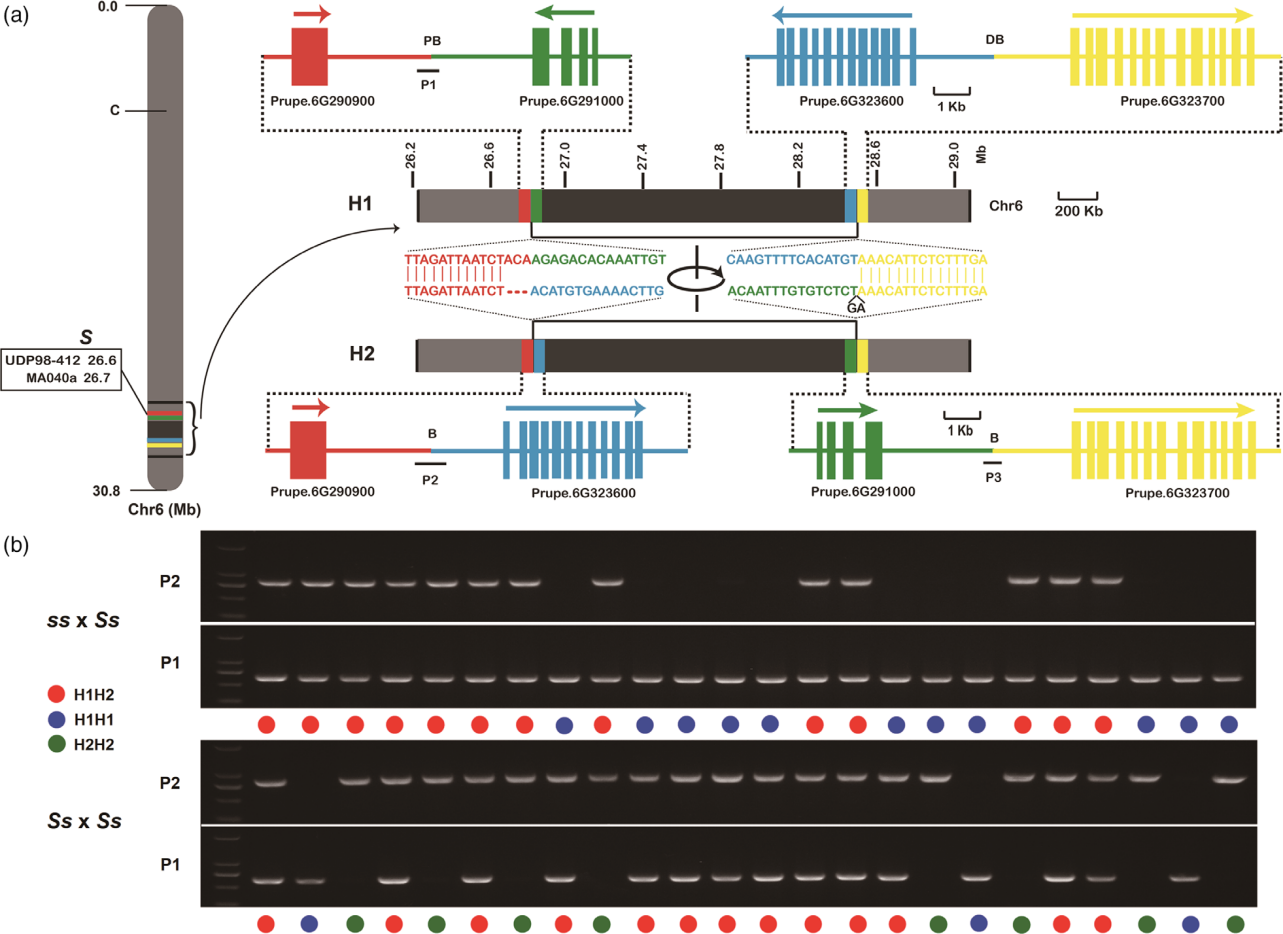
Association between chromosomal inversion and flat fruit shape in peach. a, a 1.7‐Mb inversion (grey colour) is located downstream of two SSRs, UDP98‐412 and MA040a, in the S locus co‐segregating with the fruit shape trait (left). Genes surrounding breakpoints are highlighted in different colours (right). H1 and H2 represent wild haplotype without inversion and a mutant haplotype with inversion, respectively. The H2 haplotype contained an ACA deletion and a GA insertion in the proximal and distal breakpoints, respectively. PB and DB represent proximal and distal breakpoints, respectively, and they are highlighted in a square box. P1, P2, and P3 represent PCR fragments amplified with primer pairs of P1F/P1R, P2F/P2R, and P3F/P3R, respectively, with P1 corresponding to the H1 haplotype, while P2 and P3 corresponding to the H2 haplotype. b, Genotyping of chromosomal inversions of progenies derived from ‘Shahong’ (round) × ‘Yanpan’ (flat) (top) and ‘Shennongpan’ (flat) × ‘Yanpan’ (flat) (bottom), respectively. Progenies with round‐ or flat‐shaped fruits harbour homozygous H1H1 and heterozygous H1H2 haplotypes, respectively, while progenies harbouring homozygous H2H2 haplotype bear no fruits due to incidence of abortion during early stages of fruit development.

To identify the causal mutation of the *S* locus contributing to the peach flat fruit trait, the genome of flat peach variety ‘124 Pan’ was re‐sequenced using PacBio long‐read sequencing and Illumina sequencing platforms. A total of 2.67 Gb PacBio clean data comprising 234 895 subreads were generated, with an average read length of 11.3 kb and a maximum read length of 68.5 kb (Table S1). PacBio subreads were corrected using the clean Illumina sequencing reads and then aligned to the peach reference genome v2.0 to identify presence of various structural variations (SVs). As a result, 2,165 insertions, 3180 deletions, and 82 inversions were detected (Figure 1, Table S2). Of these SVs, 394 deletions, 252 insertions, and 9 inversions were located in Chr6. Interestingly, a large chromosomal inversion of approximately 1.7 Mb in size was located immediately downstream of two SSRs, UDP98‐412 and MA040a, in the *S* locus co‐segregating with the flat fruit shape trait (Figure 2a, Figure S1). The inversion region contained 327 putative genes with similar coding sequences as predicted in the reference genome v2.0. Two haplotypes, designated H1 and H2, were found at the inversion locus in ‘124 Pan’. The H2 haplotype contained the chromosomal inversion, while the H1 haplotype had no inversion. Sequence comparison revealed that the chromosomal inversion in the H2 haplotype was flanked by a three‐nucleotide deletion and a two‐nucleotide insertion (Figure 2a).

### The 1.7‐Mb chromosomal inversion co‐segregates with the flat fruit trait in peach

To determine whether the 1.7‐Mb chromosomal inversion is associated with the flat fruit trait, three segregating F_1_ populations and a collection of peach cultivars were screened using PCR analysis. Initial analysis of a segregating F_1_ population ‘Shahong’ (round) × ‘Yanpan’ (flat) revealed the presence of two genotypes, H1H1 and H1H2, at the inversion locus (Figure 2b). Individuals with round fruits had the same genotype H1H1, while individuals with flat fruits shared an H1H2 genotype. Similar findings were observed for another F_1_ segregating population of ‘TX4C199 (round)’ × ‘5‐32’ (flat) (Table 1). By contrast, three genotypes, H1H1, H1H2, and H2H2, were detected in a F_1_ segregating population of ‘Shennongpan’ (flat) × ‘Yanpan’ (flat) (Figure 2b). While H1H1 and H1H2 were present in round and flat fruit individuals, respectively, H2/H2 corresponded to individuals bearing no fruits due to abortion during early stages of fruit development (Table 1). Subsequent genotyping of 259 peach cultivars showed that all 72 flat cultivars had the heterozygous H1H2 genotype, while all the 187 round cultivars shared the homozygous H1/H1 genotype (Figure S2, Table S3). No cultivars with the H2H2 genotype were detected, which is consistent with the finding that trees with the homozygous *SS* genotype at the *S* locus are fruitless due to early fruit abortion several weeks after flowering, thus, this genotype is selected against in peach breeding programs (Dirlewanger *et al*., 2006). These results indicated that the 1.7‐Mb chromosomal inversion co‐segregated with flat fruit shape in peach.

**Table 1:** PCR‐based identification of chromosomal inversion at the *S* locus co‐segregating with the fruit shape trait in segregating F_1_ populations and in peach cultivars

Genotype at chromosomal inversion	No. of progeny*			No. of cultivars	Fruit shape
	‘TX4C199’ × ‘5–32’	‘Shahong’ × ‘Yanpan’	‘Shennongpan’ × ‘Yanpan’		
H_1_H_1_	71	11	3	187	Round
H_1_H_2_	58	16	13	72	Flat
H_2_H_2_	0	0	8	0	N/A^†^

* ‘TX4C199’ and ‘Shahong’ are round fruit cultivars, while ‘5‐32’, ‘Yanpan’, and ‘Shennongpan’ are flat fruit cultivars.

^†^ Fruits are aborted during early stages of fruit development.

In addition, a sequencing‐based method was also conducted to identify chromosomal inversion in peach germplasm (Figure S3). Illumina genomic sequencing data of 610 different peach accessions with clear fruit shape trait and sufficient sequencing data for genotyping the inversion were retrieved from the SRA database of NCBI (Table 2, Table S4). Of these accessions, 508 were cultivars, while 102 were their wild relatives. Among the cultivars, 476 and 32 had H1H1 or H1H2 genotypes, producing round or flat fruits, respectively. Thirty‐eight cultivars, including twelve flat peaches and twenty‐six round peaches, were genotyped by both PCR‐based analysis and sequencing‐based method, and the results were well consistent.

**Table 2:** Sequencing‐based identification of chromosomal inversion at the *S* locus in peach cultivars and their wild relatives

Species	No. of accessions assessed	Genotype*		
		H1H1	H1H2	H2H2
*P. persica*	508	476	32	0
*P. ferganensis*	9	7	1	1
*P. mira*	76	76	0	0
*P. davidiana*	9	9	0	0
*P. kansuensis*	4	4	0	0
*P. mongolica*	1	1	0	0
*P. pedunculata*	1	1	0	0
*P. tangutica*	2	2	0	0

* All accessions tested with H1H1 or H1H2 genotypes produce round or flat fruits, respectively, while accessions with the H2H2 genotype bear no fruits.

For the wild relatives, chromosomal inversion was exclusively detected in *P. ferganensis* from Xinjiang Uygur Autonomous Region (Table 2, Table S4). Accessions from *P. ferganensis* contained three genotypes, H1H1, H1H2 and H2H2, corresponding to round, flat and aborting fruits, respectively. Whereas, all accessions from *P. mira*, *P. davidiana*, *P. kansuensis* Rehd., *P. mongolica*, *P. Pedunculata* and *P. tangutica* shared the same H1H1 genotype, consistent with their round fruit shape.

Taken together, the above results indicated that the 1.7‐Mb chromosomal inversion immediately downstream of the *S* locus could be the causal mutation for flat fruit shape in peach.

### Comparison of RNA‐Seq‐based transcriptome analysis between round‐ and flat‐shaped fruits of peach

**Figure 3:**
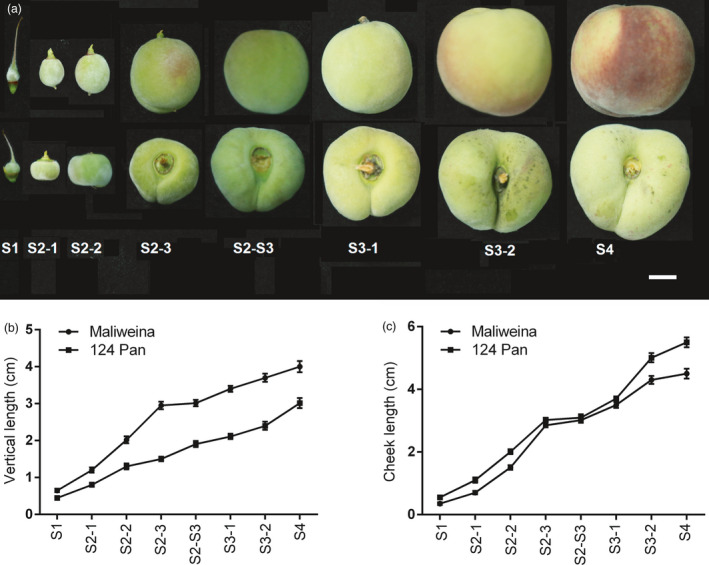
Morphological changes in fruit shape of flat and round peaches throughout fruit development. a, Fruit shapes at different developmental stages. Fruits of round peach ‘Maliweina’ and flat peach ‘124 Pan’ are present in top and bottom rows, respectively. b, Fruit vertical length. c, Fruit cheek length. Error bars correspond to SD of the mean (*n* = 20). S1, 7 days after full bloom (DAFB) at fruit set stage; S2‐1, S2‐2 and S2‐3 corresponding to 16, 24 and 32 DAFB, respectively, at the first exponential growth stage; S2‐S3, 44 DAFB at the pit hardening stage; S3‐1 and S3‐2 corresponding to 54 and 62 DAFB, respectively, at the second exponential growth stage; and S4, 70 DAFB at fruit ripening stage.

The development of stone fruits comprises of four phases (S1–S4) with two exponential growth stages. The first exponential growth stage (S2) is characterized by a rapid increase in cell division, while cell elongation plays an important role in fruit size enlargement in the second exponential growth stage (S3) (Reeve, 1959). The difference in fruit vertical diameter between flat‐shaped and round‐shaped fruits is mainly attributed to variation in cell number (Guo *et al*., 2018), and flat fruit shape is determined at the onset of flower blooming (Dirlewanger *et al*., 1998). Thus, the first exponential growth stage seems to play critical role in the fruit shape formation. To test this hypothesis, we checked the fruit vertical and cheek length of flat peach ‘124 Pan’ and round peach ‘Maliweina’ throughout fruit development (Figure 3). Round‐shaped fruits showed a faster increase in vertical length during the S2 stage than during the S3 stage, but a similar rapid increase in cheek length was observed during both S2 and S3 stages. Flat‐shaped fruits exhibited a consistent slow rate of increase in vertical length, suggesting a repression of vertical extension during the first exponential growth stage.

**Figure 4:**
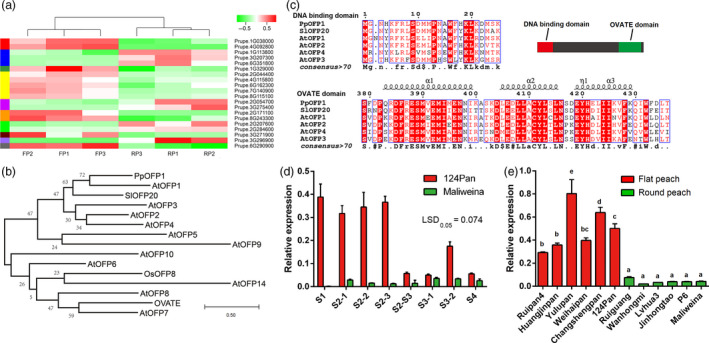
Identification of the candidate gene for the peach flat fruit trait. a, Differentially expressed genes between flat‐ and round‐shaped fruits at the S2‐2 stage. FP1 to FP3 correspond to RNA‐seq libraries of different flat peach varieties, while RP1 to RP3 correspond to RNA‐seq libraries of different round peach varieties. Different colours indicate different levels of gene expression, with red and green colours representing the highest and lowest values of gene expression, respectively. b, The DNA binding and OVATE domains of *PpOFP1* and other *OFPs* in plants. c, A phylogenetic tree derived from amino acid sequences of *OFPs* in peach, tomato, rice and *Arabidopsis*. Sequence alignment was performed using MUSCLE software, and the phylogenetic tree was constructed with MEGA‐X using maximum‐likelihood method. Accession numbers are listed as follows: *Solanum lycopersicum SlOFP20* (Solyc10g076180); *Arabidopsis AtOFP1* (AT5G01840), *AtOFP2* (AT2G30400), *AtOFP3* (AT5G58360), *AtOFP4* (AT1G06920), *AtOFP5* (AT4G18830), *AtOFP8* (AT5G19650), *AtOFP6* (AT3G52525), *AtOFP7* (AT2G18500), *AtOFP9* (AT4G04030), *AtOFP10* (AT5G22240), *AtOFP14* (AT1G79960); and *Oryza sativa OsOFP8* (Q94CV1). Numbers above the branches indicate bootstrap values of 1000 replicates. The scale bar represents 0.5 substitutions per site. d, Expression profiles of *PpOFP1* in fruits of flat peach ‘124 Pan’ and round peach ‘Maliweina’ throughout fruit development. Any difference between two group means greater than LSD_0.05_ is considered significantly different. e, Expression of *PpOFP1* in fruits at the S2‐2 stage of various peach cultivars. Error bars represent SE of three biological replicates. Different lowercase letters indicate significant difference at *P* < 0.05 based on Fisher’s Least Significant Difference (LSD) test.

To identify candidate genes controlling peach flat fruit trait, fruit samples at the S2‐2 stage (24 days after full bloom) of three flat peaches, ‘124 Pan’, ‘Zhongpan 11’ and ‘Zaolupan’, and three round peaches, ‘Maliweina’, ‘Chunli’ and ‘Wanhongmi’, were used to construct six Illumina RNA‐seq libraries, designated FP1, FP2, FP3, RP1, RP2 and RP3, respectively. Library sequencing generated 27.8, 43.2, 41.0, 41.1, 43.7 and 41.1 million clean reads with Q30 value lager than 94% for RP1, RP2, RP3, FP1, FP2 and FP3, respectively, with an accumulative size of 38.79 Gb (Table 3). The majority of the clean reads (89.6% ‐ 95.9%) were mapped to the peach reference genome v2.0 (Verde *et al*., 2017). Comparative transcriptome analysis revealed the presence of twenty differentially expressed genes (DEGs), with 14 and 6 genes either up‐ or down‐regulated, respectively, in flat‐shaped fruit samples (Figure 4a, Table 4, Figure S4). Among these DEGs, two up‐regulated (*Prupe.6G290900* and *Prupe.6G162300*) genes and one down‐regulated (*Prupe.6G351800*) gene were located on Chr6 harbouring the *S* locus for the flat fruit trait. *Prupe.6G162300* and *Prupe.6G351800* were located at the 15.3 and 30.0 Mb of Chr6, while *Prupe.6G290900* was located at 26.8 Mb near the *S* locus, tightly linked to SSR markers, UDP98‐412 (26.6 Mb), MA040a (26.7 Mb), and MA014a (27.2 Mb). In addition, 573 genes were identified within a 3‐Mb region from 26 to 29 Mb on Chr6 that covered the *S* locus and the 1.7‐Mb inversion, and only *Prupe.6G290900* showed significant difference in expression level between round and flat peaches (Table S9). Thus, *Prupe.6G290900* encoding an OVATE Family Protein, designated as *PpOFP1*, was deemed to be a candidate gene for the *S* locus.

**Table 3:** Summary of RNA‐Seq reads for three round‐ and flat‐shaped fruit libraries

Library	Raw reads	Q30	Clean reads			Total mapped reads	
			No.	Total size (bp)	GC content	No.	Percentage
RP1	55 558 884	94.66%	54 266 050	7 982 149 692	45.62%	52 026 051	95.87
RP2	44 830 188	94.69%	43 725 106	6 431 919 642	45.37%	42 239 983	96.60
RP3	41 530 428	94.07%	41 080 720	6 023 067 041	47.45%	37 312 851	90.83
FP1	43 222 558	94.69%	43 222 558	6 355 109 932	45.67%	41 331 537	95.62
FP2	41 026 286	94.94%	41 026 286	6 036 506 405	45.56%	39 342 011	95.89
FP3	41 085 348	94.69%	41 085 348	6 021 881 758	47.63%	36 816 394	89.61

**Table 4:** List of DEGs between flat‐ and round‐shaped fruit RNA‐Seq libraries

	DEG	RNA‐Seq library*						Physical location	Annotation
		RP1	RP2	RP3	FP1	FP2	FP3		
Up‐regulated	Prupe.4G092800	0.00	0.00	0.03	12.41	6.90	14.63	Pp04: 4658153‐4662306	Cyclic nucleotide gated channel 1
	Prupe.2G171100	0.03	0.00	0.00	7.92	8.21	1.58	Pp02:21820726‐21826349	SKP1‐like 20
	Prupe.1G038000	32.89	65.86	26.71	406.78	178.70	414.22	Pp01:2657410‐2658362	Unknown
	Prupe.1G329000	16.28	11.43	3.14	268.07	53.62	66.08	Pp01:31263175‐31264260	MLP‐like protein 28
	Prupe.8G243300	0.81	4.13	0.37	19.76	31.58	13.66	Pp08:21231807‐21234801	Phenazine biosynthesis PhzC/PhzF protein
	Prupe.2G044400	0.10	0.00	0.00	2.33	0.99	2.04	Pp02:4954292‐4962100	Leucine‐rich repeat protein kinase
	Prupe.6G290900	2.20	0.84	5.93	51.36	60.15	95.02	Pp06:26842544‐26844228	OVATE family protein
	Prupe.7G140900	3.46	3.70	2.79	13.44	18.47	7.21	Pp07:15637802‐15639226	Methyl esterase
	Prupe.3G271900	0.41	0.37	0.00	1.47	14.21	7.03	Pp03:25242674‐25245002	PHB domain‐containing protein
	Prupe.6G162300	3.25	6.21	1.15	12.92	15.09	8.86	Pp06:15301313‐15305769	Unknown
	Prupe.8G115100	2.74	3.58	2.33	9.24	10.69	5.56	Pp08:14248943‐14251302	Oxygenase superfamily protein
	Prupe.4G115800	52.65	55.43	31.95	119.44	117.90	67.47	Pp04:6257151‐6259483	Thioredoxin O1
	Prupe.3G296900	24.19	14.00	2.26	0.00	0.05	0.00	Pp03:26369333‐26372217	Unknown
	Prupe.6G351800	4.86	0.81	2.83	0.09	0.00	0.02	Pp06:299764090‐29981222	Unknown
Down‐regulated	Prupe.3G275400	1.48	6.11	0.73	0.00	0.00	0.00	Pp03:25387922‐25391450	Unknown
	Prupe.2G054700	10.44	10.52	1.69	0.87	1.30	0.53	Pp02:6460427‐6466614	NB‐ARC domain protein
	Prupe.3G207300	5.11	2.01	1.64	0.00	0.00	0.00	Pp03:21395237‐21396416	pre‐mRNA splicing factor‐related
	Prupe.1G113800	21.68	11.39	21.79	3.38	4.30	0.92	Pp01:9041293‐9042145	Pectin methylesterase inhibitor
	Prupe.2G207600	25.39	68.78	59.96	11.48	8.54	10.97	Pp02:24134001‐24135971	Hydroxyisobutyryl‐CoA hydrolase
	Prupe.2G284600	1.74	1.92	1.3	0.48	0.34	0.34	Pp02:28180402‐28183113	Tetratricopeptide repeat protein

* The values in the columns below the libraries represent the TPM values.

The *PpOFP1* gene has a coding sequence of 1326 bp and encodes a putative OVATE family protein of 441 amino acid residues. PpOFP1 contains a putative transcriptional repressor OVATE domain in the C‐terminus and a DNA binding domain in the N‐terminus, both of which represent the conserved features of the *OFP* gene family (Figure 4b). In addition, *PpOFP1* is phylogenetically related to both *AtOFP1* and *SlOFP20* (Figure 4c), which are negative regulators of cell elongation in *Arabidopsis* (Wang *et al*., 2007) and ovary development in tomato (Wu *et al*., 2018), respectively. Thus, *PpOFP1* is likely to repress fruit elongation, resulting in flat fruit shape in peach.

### The high expression of the candidate *PpOFP1* is associated with the flat fruit trait in peach

The expression profile of *PpOFP1* in flat‐ and round‐shaped fruits throughout the whole development was investigated using qRT‐PCR (Figure 4d). *PpOFP1* showed higher levels of expression in fruits of ‘124 Pan’ than in fruits of ‘Maliweina’ throughout all stages of fruit development. The expression of *PpOFP1* exhibited the highest levels in fruits of ‘124 Pan’, but extremely low or nearly undetectable levels in fruits of ‘Maliweina’ at the fruit set (S1) and S2 stage. The expression levels of *PpOFP1* in flat‐shaped fruits were 11.7‐, 21.2‐, and 27.7‐fold higher than those in round‐shaped fruits at S2‐1, S2‐2, and S2‐3 stages, respectively. Moreover, RNA in situ hybridization was conducted to explore the localization of *PpOFP1* mRNA in fruits of ‘124 Pan’ at the S2‐2 stage. The mRNA of *PpOFP1* was found to be predominantly localized in vigorous cell division zones of epicarp and endocarp (Figure S7), suggesting its potential inhibitory effect on cell proliferation. These findings were consistent with the above‐mentioned result of repression of vertical extension in flat‐shaped fruits at the S2 stage. In addition, the expression levels of *PpOFP1* showed significant decreases in flat‐shaped fruits at the S3 stage and at ripening stage (S4), except the S3‐2 stage where moderate levels of expression were detected. Overall, expression of *PpOFP1* throughout fruit development showed a good synergy with fruit shape development.

Expression of *PpOFP1* was further investigated in fruits at the S2‐2 stage of six flat and six round peach accessions (Figure 4e). *PpOFP1* was highly expressed in fruits of flat peach cultivars, whereas it was lowly expressed in fruits of round peach cultivars, thus further supporting the above‐mentioned association between *PpOFP1* expression and peach fruit shape. Based on the above findings, it is proposed that *PpOFP1* is likely to play a repressive role in fruit vertical extension, with high expression levels leading to flat fruit shape in peach.

### Sequence polymorphisms in genomic region and upstream of *PpOFP1*

**Figure 5:**
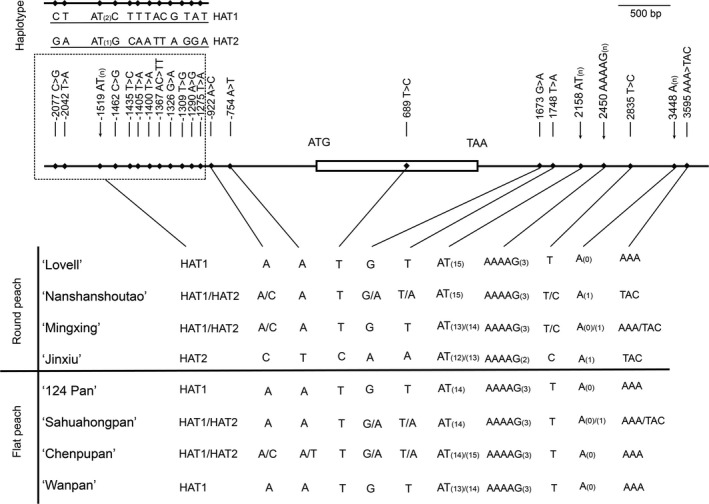
Genomic DNA sequence variation of *PpOFP1* in peach. a, Polymorphic sites at promoter, genic and downstream regions of the *PpOFP1* gene. The numbers below polymorphic sites represent physical locations in base pairs relative to the start codon. HAT1 and HAT2 represent two haplotypes in the promoter region of *PpOFP1*. b, Genotyping polymorphic sites of the *PpOFP1* gene in flat and round peach varieties. DNA sequences for ‘124 Pan’ and ‘Lovell’ were retrieved from Illumina whole‐genome resequencing data and the peach reference genome v2.0, respectively. Whereas, DNA sequences for flat peach accessions, ‘Sahuahongpan’, ‘Chenpupan’ and ‘Wanpan’, and round peach accessions, ‘Nanshanshoutao’, ‘Mingxing’ and ‘Jinxiu’, were recovered using PCR‐based direct sequencing method.

To detect genomic sequence variation of *PpOFP1*, genomic sequencing reads of ‘124 Pan’ were mapped to the reference genome v2.0 of round peach ‘Lovell’. A polymorphic AT SSR motif, 858 bp downstream of the stop codon of *PpOFP1*, was detected, but with no polymorphic sequences in both coding and promoter regions. Subsequently, high‐fidelity PCR was conducted to amplify whole‐genomic DNA sequences of *PpOFP1*, including 2.2‐Kb upstream and 2.4‐Kb downstream sequences, in three flat and three round peach accessions. Sequence comparisons among peach accessions revealed the presence of twenty‐two polymorphic sites, with twelve forming two haplotypes, HAT1 and HAT2 (Figure 5a). However, all these variants, including the (AT)_n_ motif, showed no association with the fruit shape trait (Figure 5b).

**Figure 6:**
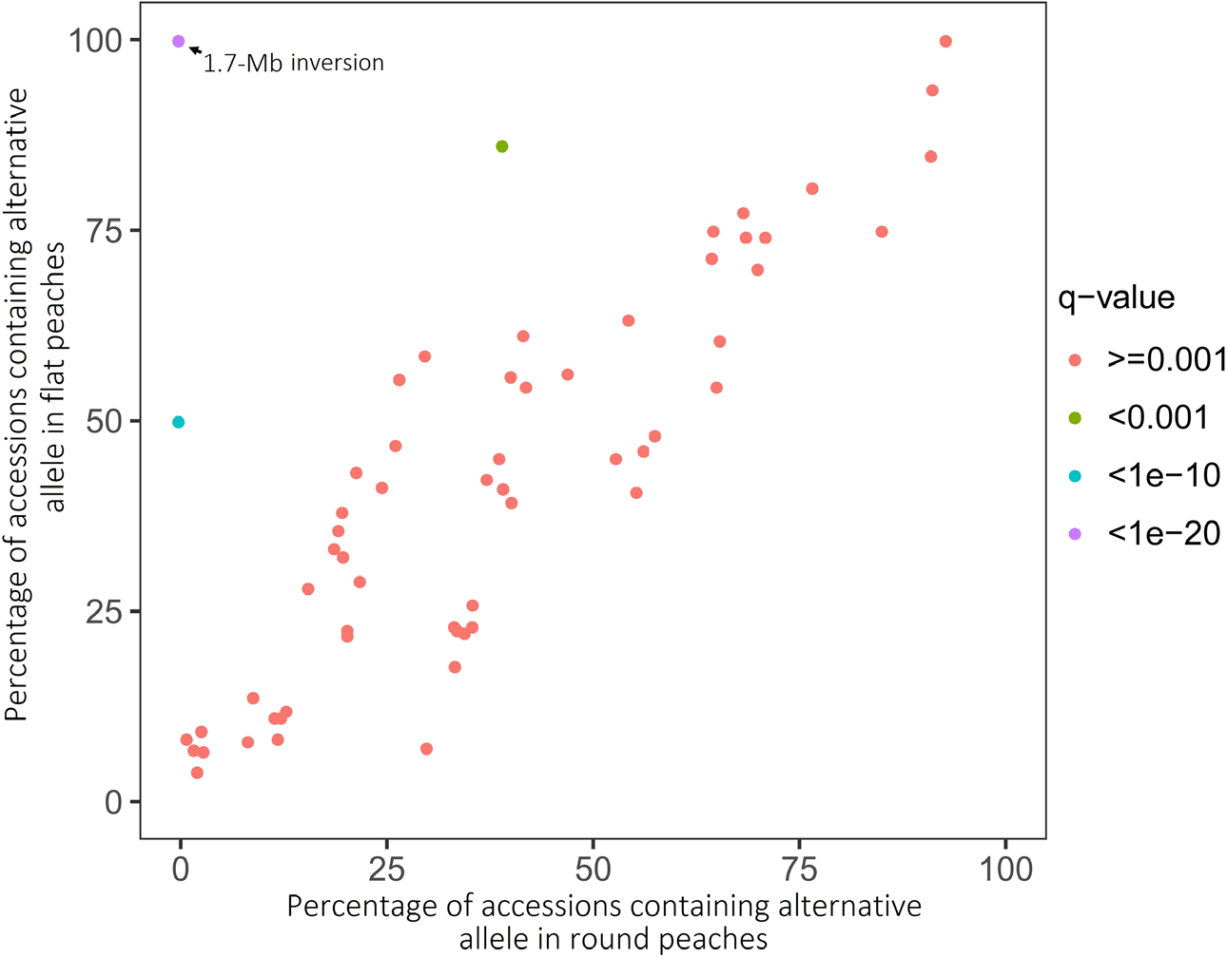
Comparison of alternative allele frequency between flat and round peaches. The significant level was calculated using Fisher test and corrected using false discovery rate (FDR, q‐value). Circle dots indicate alternative alleles, which are different from those found in the reference genome v2.0 of round peach ‘Lovell’.

Two SSRs, UDP98‐412 and MA040a, tightly linked to the *S* locus, are located 224.7 and 120.4 Kb, respectively, upstream of the start codon of *PpOFP1*. We investigated whether genomic sequence variation upstream of *PpOFP1* might be associated with the fruit shape trait. Given the fact that flat peach cultivar is characterized by heterozygosity in the *S* locus (Dirlewanger *et al*., 2006), we screened heterozygous loci in an approximately 225‐Kb region upstream of the *PpOFP1* gene in ‘124 Pan’. As a result, a total of 63 heterozygous SNPs were identified (Table S5). These SNPs were used for genotyping of 517 peach cultivars and *P. ferganensis* accessions, including 34 round peach accessions and 483 flat peach accessions, based on their genomic resequencing data retrieved from NCBI (Table S6). To detect whether alterative alleles that is different from those in the reference genome of round peach, ‘Lovell’ are related to flat fruit shape, alternative allele frequency in round and flat peaches were calculated (Table S7). Frequency of two alternative alleles at positions 26,666,382 (blue dot) and 26,838,110 (green dot), respectively, showed a significant difference between round and flat peaches (Figure 6). However, these two alternative alleles were either absent in some flat peaches or present in both flat and round peaches, suggesting that they are unlikely the causal mutations for the flat fruit trait. By contrast, frequency of the 1.7‐Mb chromosomal inversion was significantly different between round and flat peaches, with its exclusive presence in flat peaches, but absent in round peaches (Figure 6).

Taken together, these results suggested that the 1.7‐Mb chromosomal inversion is responsible for activation of *PpOFP1* in flat‐shaped fruits of peach. Thirteen putative *cis*‐elements were identified in a 1.5‐kb region downstream of the PB site (Table S10). Since auxin is well known to stimulate cell elongation via increasing wall extensibility (Velasquez *et al*., 2016), the D4 AuxRE could be an enhancer‐like element that induced the activation of *PpOFP1* in flat‐shaped fruits.

### Ectopic overexpression of *PpOFP1* negatively affects leaf and silique growth in *Arabidopsis*

**Figure 7:**
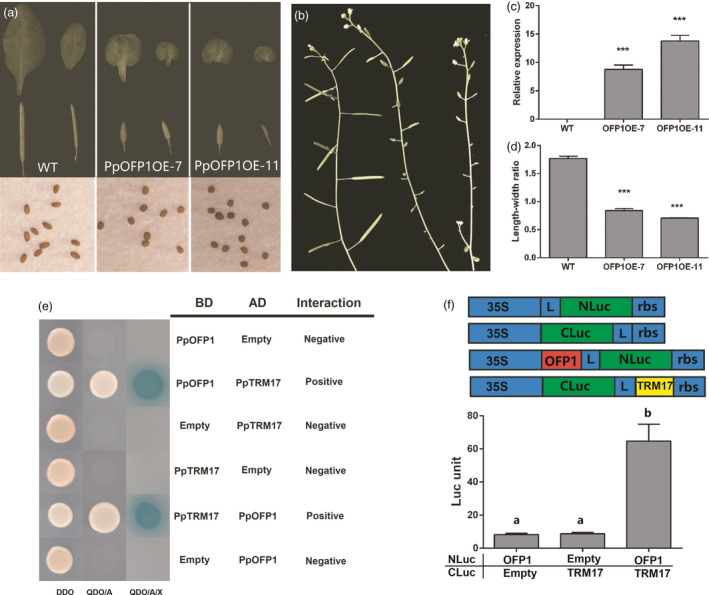
Functional analysis of the *PpOFP1* gene. a, Leaves and siliques of eight‐week‐old *Arabidopsis* transgenic lines overexpressing *PpOFP1* and the wild type (Col‐0). Transgenic lines produced oval‐shaped leaves and shortened siliques enclosing round‐shaped seeds. b, Expression of *PpOFP1* in leaves of transgenic lines and the wild type. Error bars represent SE of three biological replicates. ***, *P* < 0.001 (Student’s *t*‐test). c, Comparison of the leaf length‐width ratio between transgenic lines and the wild type. d, Siliques on branch of eight‐week‐old transgenic lines and the wild type. e, Analysis of interaction between PpOFP1 and PpTRM17 using a yeast two‐hybridization system. f, Analysis of interaction between PpOFP1 and PpTRM17 using split firefly luciferase complementation assay in young *Nicotiana benthamiana* leaves. The error bars show ± SE of four biological replicates. Different lowercase letters indicate significant difference at *P* < 0.01 based on Fisher’s LSD test.

To validate whether *PpOFP1* functions as a negative regulator, its full‐length coding sequences under the control of the CaMV35S promoter were transferred into the *Arabidopsis* (Figure 7a, d). Real‐time PCR assay showed that *PpOFP1* was highly expressed in transgenic lines (Figure 7b). The leaves of transgenic lines were oval‐shaped and smaller in size compared with the wild type (Figure 7a). The leaf length‐width ratios of transgenic lines were significantly lower than those of the wild type (Figure 7c). Moreover, transgenic lines produced shortened siliques, but with no obvious change in circumference (Figure 7a, d), which is quite similar to shortened vertical length and normal cheek length observed in flat peaches. Additionally, siliques of transgenic lines enclosed round‐shaped seeds which are shorter than the seeds of wild‐type *Arabidopsis* (Figure 7a). These results suggested that *PpOFP1* functions as cell elongation repressor, thus, participating in regulation of fruit shape in peach.

### PpOFP1 is able to interact with TONNEAU1 recruiting motif protein (TRM) in peach

As OFPs are known to interact with TONNEAU1 Recruiting Motifs (TRMs) to regulate plant organ shape (Li *et al*., 2011; Wu *et al*., 2018), the peach genome was screened to identify *TRM* homologs. As a result, 12 *PpTRM* genes were identified, including three members, *Prupe.8G209800*, *Prupe.2G170700* and *Prupe.6G315200*, showing high levels of expression in both round‐ and flat‐shaped fruits (Figure S5A). Phylogenetic analysis revelled that *Prupe.8G209800*, designated *PpTRM17*, is closely related to *SlTRM17* (Figure S5B). Thus, it was selected to test its interaction with *PpOFP1*. Both yeast two‐hybridization (Figure 7e) and firefly luciferase complementation assay (Figure 7f) demonstrated that PpOFP1 could interact with PpTRM17 *in vivo*. In addition, physical interaction was detected between PpOFP1 and SlTRM17/20a and between SlOFP20 and PpTRM17 (Figure S8), suggesting that *PpOFP1* might have the same function as *SlOFP20* in tomato.

## Discussion

The flat fruit trait in peach is a qualitative trait controlled by a single dominant *S* locus at the bottom of Chr6 (Dirlewanger *et al*., 1999; Lesley, 1940). Several candidate genes for the *S* locus have been reported. *PpCAD1* was initially deemed to be a candidate gene as an A to T substitution in its intron co‐segregates with the flat fruit trait (Cao *et al*., 2016). However, the expression of *PpCAD1* showed no differences between flat and round fruits at the early stages of development, which is in conflict with the fact that the fruit shape starts to form at early fruit development (Guo *et al*., 2018). Later, a *LRR‐RLK* gene was assumed to be the candidate gene for the flat fruit trait (Lopez‐Girona *et al*., 2017). The *LRR‐RLK* gene can interact with *CLAVATA2* to control stem cell population size, and its null mutation may cause a decrease in fruit vertical length (Lopez‐Girona *et al*., 2017). However, this hypothesis is in contrast with the finding that the *LRR‐RLK* gene is not associated with flat fruit phenotype in Chinese peach cultivars (Guo *et al*., 2018).

In this study, *PpCAD1* and *PpLRR‐RLK* genes showed similar expression levels in fruits at the first exponential growth stage between flat and round peaches (Figure S9). However, only *PpOFP1* out of the genes within the *S* locus showed different expression profiles between flat‐ and round‐shaped fruits. Analysis of a previously reported transcriptome dataset (Guo *et al*., 2018) revealed that the *PpOFP1* gene showed higher levels of expression in flat‐shaped fruits of ‘Zao Huang Pan Tao’ at both flowering (S1) and early stage of fruit development (S2) than those in round‐shaped fruits of ‘Zhong Tao Hong Yu’, with 21.7‐ and 22.7‐fold changes in FPKM values, respectively (Figure S6). The failure to detect *PpOFP1* as candidate gene for the *S* locus in previous studies might be due to similar expression levels in flat‐ and round‐shaped fruits at early stages of fruit ripening. Here, *PpOFP1* was also highly expressed in flat‐shaped fruits at the juvenile stage, but significantly decreased in expression level at the second exponential growth and ripening stages. This is consistent with the finding that flat fruit shape is determined at fruit set and the first exponential growth stages, in which cell number in vertical diameter showed a great variation between flat‐ and round‐shaped fruits (Dirlewanger *et al*., 1998; Guo *et al*., 2018). Therefore, genes showing differential expression patterns between flat‐ and round‐shaped fruits at fruit set and the first exponential growth stages, but with similar expression profiles at the second exponential growth and ripening stages should be selected as candidates for the flat fruit trait. In our comparative transcriptome analysis, only *PpOFP1* met these criteria. The ectopic overexpression of *PpOFP1* in *Arabidopsis* resulted in oval‐shaped leaves and shortened siliques, which is similar to previously reported results of functional analysis for its ortholog *AtOFP1* (Hackbusch *et al*., 2005). These results suggest that *PpOFP1* is a strong candidate for the flat fruit trait in peach. In addition, *PpOFP1* is able to interact with *PpTRM17*, which suggests that peach fruit shape is likely controlled by the OFP‐TRM module, a common mechanism underlying organ shape in plants (Wu *et al*., 2018). It is important to note that *PpTRM17* is a homolog of *SlTRM17* that promotes the elongation of tomato fruit. Thus, we speculate that *PpTRM17* promotes the elongation of fruit, while *PpOFP1* plays an antagonistic role in peach. The relative expression levels of *PpOFP1* and *PpTRM17* are critical in controlling the ultimate shape of a peach fruit. Additionally, one (*Prupe.1G113800*) out of the six down‐regulated DEGs between round and flat peaches encodes a pectin methylesterase inhibitor protein. Given the role of pectin in the cell division process, it is worthy of further study to ascertain whether *Prupe.1G113800* is a downstream target of *PpOFP1*.

Chromosomal inversion is a kind of structural variation produced by reinsertion of segment bounded by the breakpoints in the reversed orientation. Chromosomal inversion may cause genomic disorders associated with side effect of breakpoints, such as disruption of the open reading frame, or separation of transcription units from *cis*‐acting regulatory elements resulting in changes in expression levels (Feuk *et al*., 2006; Kleinjan and Heyningen, 1998). In this study, the 1.7‐Mb chromosomal inversion downstream of *PpOFP1* showed an association with the fruit shape trait in 727 peach cultivars. Therefore, the elevated expression level of the *PpOFP1* gene by the 1.7‐Mb chromosomal inversion event in flat peaches could be explained by the new combination of downstream *cis*‐element and transcription unit. Similar phenomenon has also been reported in humans and animals. A large chromosomal inversion approximately 70‐kb downstream of the *KIT* gene probably disrupts a regulatory element of the gene, resulting in the tobiano spotting pattern in German horse breeds (Haase *et al*., 2008). A chromosomal inversion 200‐kb downstream of a well known language gene, *FOXP2*, causes a decrease in its expression, leading to language disorder in humans (Moralli *et al*., 2015). In addition, illegitimate DNA end joining at chromosomal DNA double‐strand breaks (DSBs) via the non‐homologous end joining (NHEJ) pathway is an important mechanism underlying chromosomal inversion (Mizukami *et al*., 2014; Nicholas *et al*., 2018). NHEJ is a highly error‐prone repair and often induces small insertions and deletions at the site of the DSB. Interestingly, the H2 haplotype contained a three‐base‐pair deletion in the proximal breakpoint and a two‐base‐pair insertion in the distal breakpoint. Thus, the 1.7‐Mb inversion was probably induced by chromosomal DNA DSB along with illegitimate joining of DNA ends through the NHEJ pathway.

Chromosomal inversion plays an important role in suppressing recombination in heterozygotes (Kirkpatrick, 2010). Large inversion heterozygotes often have sister chromosome pairing problems in the inverted region, leading to a reduction of the recombinant frequency. As a consequence, a slower decay of LD can be expected in the large inverted chromosomal region (Hoffmann and Rieseberg, 2008). This could explain the presence of a large LD block at the *S* locus, resulting in DNA markers or genes, including *PpCAD1* and *PpLRR‐RLK*, within or close to the inverted region co‐segregating with the flat peach trait.

The domestication of peach was thought to occur initially in the region of Northwest China between the Tarim basin and the north slopes of the Kunlun Shan Mountains (Faust and Timon, 1995). In this study, our results show that the 1.7‐Mb chromosomal inversion in the *S* locus is present in *P. ferganensis*, but not in other wild relatives. *P. ferganensis* is native to Xinjiang province of Northweste China and the Ferghana valley on the west side of the Tarim basin in Central Asia (Scorza and Okie, 1990). New research shows that *P. ferganensis* is an intermediate genome in peach domestication as it is phylogenetically closely related to cultivars (Cao *et al*., 2014; Verde *et al*., 2013). Hence, our study provides additional evidence to support the assumption that peach domestication occurred in the region of Northwest China. The peach flat fruit trait is likely to have originally occurred in *P. ferganensis* and was then introduced to peach cultivars.

## Methods

### Plant materials

All peach accessions used in this study are maintained at Wuhan Botanical Garden of the Chinese Academy of Sciences (Wuhan, Hubei Province) and Jiangsu Academy of Agricultural Sciences (Nanjing, Jiangsu Province). All accessions can be divided into either round or flat peaches, with flat peaches showing a flattened shape (cheek length/vertical length >1.5) in contrast to ordinary rounded peaches (cheek length/vertical length < 1.2). Fruit samples of round peach ‘Maliweina’ and flat peach ‘124 Pan’ were collected at the following developmental stages: S1 (fruit set), S2 (the first exponential growth), S2‐S3 (pit hardening), S3 (the second exponential growth) and S4 (fruit ripening). Fruit samples of five flat and five round peach cultivars were collected at the S2 stage. Each sample consisted of three biological replicates, and each replicate containing at least five fruits was collected from a single tree. Fruits were cored, cut into pieces, immediately frozen in liquid nitrogen and then stored at −80 °C until use.

### Construction of RNA‐Seq libraries for Illumina sequencing and data analysis

Total RNA was extracted using Trizol reagent, and RNA concentration was measured using Thermo Scientific NanoDrop 2000. RNA integrity was assessed using the RNA Nano 6000 Assay Kit of the Agilent Bioanalyzer 2100 system (Agilent Technologies, CA). Approximately 1 μg of total RNA was used for RNA library construction. Sequencing libraries were generated using a NEBNext Ultra RNA Library Prep Kit for Illumina (NEB, USA) according to the manufacturer’s instructions. Briefly, the library fragments were purified with an AMPure XP system (Beckman Coulter, Beverly) to preferentially select cDNA fragments of 240 bp in length. PCR reaction was conducted with NEB Phusion High‐Fidelity DNA polymerase, and PCR products were purified and checked on the Agilent Bioanalyzer 2100 system. The clustering of the index‐coded samples was performed on a cBot Cluster Generation System using TruSeq PE Cluster Kit v4‐cBot‐HS (Illumia) following the manufacturer’s instructions. After cluster generation, the libraries were sequenced on an Illumina platform to generate paired‐end reads.

Raw data in FASTQ format files were processed to remove adapter and low quality reads. To assess the quality of clean data, Q20, Q30 and GC content, as well as sequence duplication levels, were determined. Clean data were mapped to the peach reference genome V2.0 (Verde *et al*., 2013; Verde *et al*., 2017) using Hisat2 software (Kim *et al*., 2015). Transcripts were assembled and merged using StringTie software (http://ccb.jhu.edu/software/stringtie/). Gene expression levels were estimated by Transcripts Per Million (TPM) reads using RSEM software (http://deweylab.github.io/RSEM/). Differential expression analysis was performed using the DESeq2. Genes with an adjusted log2 fold change (FC) > 2 and false discovery rate (FDR) < 0.1 were assigned as differentially expressed. The hierarchical cluster analysis of the DEGs was conducted using RSEM software. Function annotation of DEGs was retrieved from the database of the peach reference genome V2.0 (https://www.rosaceae.org/). The RNA‐seq data have been deposited in NCBI Bio‐Project with the accession number PRJNA588956.

### Quantitative real‐time PCR (qRT‐PCR)

Total RNA extraction was conducted using Total RNA Rapid Extraction Kit (Zomanbio, Beijing, China), and RNase‐free DNase I (NEB) was used to eliminate any genomic DNA contamination. First‐strand cDNA synthesis was performed using PrimeScript^TM^ RT reagent Kit with gDNA Eraser (Takara, Dalian, China). qRT‐PCR was conducted using SYBR® Premix Ex Taq™ II (Takara Bio, Inc.), with the following amplification program: one cycle of 30 s at 95 °C, followed by 40 cycles of 5 s at 95 °C and 34 s at 60 °C. A previously reported translation elongation factor gene *PpTEF2* was selected as the internal control (Tong *et al*., 2009). Relative gene expression levels were calculated using the formula 2^−∆∆Ct^. Three biological replicates were conducted for each sample. Sequences of the primers used for qRT‐PCR are listed in Table S8.

### Cloning of the promoter, genic, and downstream regions of *PpOFP1*

Two pairs of primers, Pro1F/Pro1R and Pro2F/Pro2R, were designed to amplify two overlapped fragments spanning a 2.2‐kb promoter region upstream of the start codon using the Ex Taq enzyme (Takara, Dalian, China). PCR products were cloned using the pEASY‐T1 Cloning Kit (TRANSGEN BIOTECH, Beijing), and then subjected to Sanger sequencing to identify DNA polymorphisms. Similar methods were used to isolate both genic and downstream regions.

### Whole‐genome resequencing of flat peach ‘124 Pan’ using Pacbio Sequel system

Genomic DNA was extracted from young leaves using the CTAB protocol (Porebski *et al*., 1997), and DNA quality was evaluated by pulsed‐field gel electrophoresis and a Qubit fluorometer. Genomic DNA was sheared with a 26G Needle and DNA fragments >20 Kb were selected with the BluePippin system. Following blunt‐end ligation, adenylation of 3′ ends of DNA fragments, and adaptor ligation, the final library was sequenced using PacBio Biosciences Sequel third‐generation sequencing platform. Raw data in FASTQ format files were processed using SMRTlink v4.0 software (PacBio) with parameters: minLength = 50 and minReadScore = 0.8. The quality of sequencing data was confirmed by the assessment of the N50 value, the average length of the polymerase reads, and Subreads.

In addition, genomic DNA of ‘124 Pan’ was also re‐sequenced using an Illumina HiSeq X Ten Sequencing System, and clean data were used to correct the PacBio clean data using the MaSuRCA software (Zimin *et al*., 2013). Corrected PacBio clean data were then mapped and reordered according to the peach reference genome v2.0 using ngmlr (Sedlazeck *et al*., 2018) and SAMTOOLS software (Li *et al*., 2009), respectively. SNPs and InDels were identified using SAMTOOLS software. Sequence annotation was conducted using the ANNOVAR software (Kai *et al*., 2010). Structural variations, including insertion, deletion, inversion, intra‐chromosomal translocation (ITX), and inter‐ chromosomal translocation (CTX), were detected using the BreakDancer software (Fan *et al*., 2014). Copy number variations (CNVs) were called using Sniffles (https://github.com/fritzsedlazeck/Sniffles). The Pacbio and Illumina sequence data have been deposited in NCBI Bio‐Project with the accession number PRJNA588956.

### Investigation of the 1.7‐Mb chromosome inversion at the *S* locus in peach accessions

PCR‐ and sequence‐based methods were used to identify genotypes at the *S* locus. For PCR‐based method, a pair of primers, P1F/P1R, located at inversion breakpoint proximal to *PpOFP1*, was designed to assay wild haplotype with no inversion, whereas, two pairs of primers, P2F/P2R and P3F/P3R, located at proximal and distal inversion breakpoints, respectively, were designed to assay mutant haplotype with inversion. PCR amplification was conducted using the Golden PCR Mix (Tsingke, Beijing). After validation by direct sequencing, these three pairs of primers were used to genotype peach cultivars.

For sequencing‐based method, Illumina genomic resequencing data of peach cultivars and their wild relatives were retrieved from the SRA database of NCBI. After cleaning, the raw reads of each accession were mapped to sequences of wild type and inverted haplotypes at the *S* locus to identify reads spanning proximal and distal breakpoints (Figure S3). The chromosomal inversion was deemed to occur if both breakpoint‐spanning and split reads were identified from inverted and wild‐type haplotypes, respectively.

### *Arabidopsis* transformation

Whole‐coding region of *PpOFP1* was amplified and cloned into the plant overexpression binary vector pSAK277. *Arabidopsis* transformation was conducted using the floral dip method according to the previous reports (Clough and Bent, 1999).

### RNA in situ hybridization

Young fruits of ‘124 Pan’ at S2 stage (24 DAFB) were fixed with 45% ethanol, 5% formalin, 5% acetic acid for two days at 4 °C. A digoxigenin‐labelled probe (5‐dig‐AGUCUCGGUAGCAAGAACACGGUCACACCUG‐3) was synthesized for hybridization. The hybridization was performed according to a previous report (Zanon *et al*., 2015). After visualization in NBT solution, the samples were then incubated in nuclear fast red solution.

### Yeast two‐hybrid assay

The full‐length coding sequence of *PpOFP1* was amplified using a pair of primers, OFP1BDF, and OFP1BDR. PCR products were digested with NdeI and BamHI, and inserted into Y2H vector *pGBKT7* as the bait. The full‐length sequence of *PpTRM17* was inserted into *pGADT7* via homologous recombination to generate the prey vector *PpTRM17*‐*pGADT7* using the ClonExpressII One Step Cloning Kit (Vazyme, Nanjing, China) according to the manufactures’ instructions. The yeast two‐hybrid assay was conducted using the Matchmaker® Gold Yeast Two‐Hybrid System (Clontech, Japan). The bait, prey and empty vectors were transformed into yeast strain ‘Y2Hgold’ using the Frozen‐EZ Yeast Transformation II kit (ZYMO RESEARCH). Yeast cells were grown on the DDO (SD‐Trp‐Leu), QDO/A (SD‐Trp‐Leu‐Ade‐His + AbA) and QDO/A/X (SD‐Trp‐Leu‐Ade‐His + AbA + X‐α‐Gal) medium, respectively. Photographs were taken after 3 days following incubation.

### Split firefly luciferase complementation assay

Split Firefly luciferase complementation assay was conducted according to a previous report (Chen *et al*., 2008). Briefly, whole‐coding region of *PpOFP1* (without stop codon) was amplified and inserted into the binary vector pCambia1300NLuc, while the coding sequences of PpTRM17 were cloned and inserted into the binary vector pCambia1300CLuc. These constructs were individually transformed into *Agrobacterium* strain GV3101 and incubated at 28 °C for 48 h. The confluent bacterium was resuspended in the infiltration buffer containing 10 mm 2‐(N‐morpholine)‐ethanesulphonic acid (pH = 5.7), 10 mm MgCl2 and 200 μm acetosyringone and incubated at room temperature for 2 h before infiltration. *Agrobacterium* cultures containing the NLuc and CLuc cassettes were mixed in a 1:1 ratio and injected into young leaves of 3‐week‐old Nicotiana benthamiana seedlings. Leaf discs (2 cm in diameter) adjacent to the infiltration site were punched to measure firefly luciferase (luc) activity using Steady‐Glo Luciferase Assay System (Promega) on an Infinite M200 luminometer (Tecan, Mannerdorf, Switzerland).

## Conflicts of interest

The authors declare that there is no conflict of interests.

## **Author** **contributions**

YH and HZ conceived and designed the experiments. FR, RM, JZhang, SX and JZhao collected the plant samples. HZ and QY performed the experiments. LG, LL, AZ, XZ and WZ conducted bioinformatics analysis. YH and HZ wrote the paper. CO, MY, SSK and QJ revised the manuscript.

## Supporting information


**Fig. S1** The PacBio subreads surrounding the breakpoints of the 1.7‐Mb chromosomal inversion. Subreads that were split at the proximal breakpoint (PB, A) and the distal breakpoint (DB, B) were labeled with arrows.
**Fig. S2** A schematic diagram of genotyping chromosomal inversions in different peach cultivars.
**Fig. S3** Schematic diagram for identification of H1 and H2 haplotypes at the S locus based on Illumina HiSeq reads.
**Fig. S4** The volcano and scatter plots of DEGs. Genes with an adjusted log2 fold change (FC)> 2 and false discovery rate (FDR) < 0.05 were deemed as differentially expressed.
**Fig S5** *PpTRM* genes in the peach genome. A, Expression of *PpTRM* genes in flat‐ and round‐shaped fruits at the S2‐2 stage.
**Fig S6** Expression levels of *PpOFP1* at three different fruit developmental stages derived from a previous RNA‐Seq study (Guo et al., 2018). ‘Zao Huang Pan Tao’ and ‘Zhong Tao Hong Yu’ are flat and round peach cultivars, respectively.
**Fig. S7** Analysis of RNA in situ hybridization for localization of *PpOFP1* mRNA in fruit of ‘124 Pan’ at the S2‐2 stage.
**Fig. S8** Analysis of interaction between OFPs and TRMs using the yeast two‐hybrid system.
**Fig. S9** Expression of *PpLRR‐RLK* and *PpCAD1* in fruits at the S2‐2 stage of various peach cultivars.

**Table S1** Overview of the PacBio‐Seq libraries.
**Table S2** SVs identified in the genome of cv. 124 Pan compared with the ‘Lovell’ reference genome.
**Table S3** Identification of genotypes at the S locus in peach germplasm using PCR‐based method (red color indicates presence of different haplotypes).
**Table S4** Identification of genotypes at the S locus in peach germplasm using the sequence‐based method.
**Table S5** Heterozygous SNPs detected in an approximately 225‐Kb upstream region of *PpOFP1* in flat peach cv. 124 Pan.
**Table S6** Sequencing‐based genotyping of peach accessions based on 63 heterozygous SNPs in the S locus.
**Table S7** The presence of alternative allele in flat and round peach accessions.
**Table S8** Sequences of primers used for cloning and qRT‐PCR.
**Table S9** Expression of genes within a 3‐Mb region between 26 and 29 Mb on Chr6.
**Table S10** Putative cis‐elements in a 1.5‐kb region downstream of the PB site of the 1.7‐Mb inversion on Chr6.
