# Supplementary material for: A 1.7‐Mb chromosomal inversion downstream of a PpOFP1 gene is responsible for flat fruit shape in peach
Source: Plant Biotechnol J. 2020 Aug 17;19(1):192–205. doi: 10.1111/pbi.13455 (PMC7769229; doi:10.1111/pbi.13455)
Supplement: Supplementary file 1 — Fig. S1 The PacBio subreads surrounding the breakpoints of the 1.7‐Mb chromosomal inversion. Subreads that were split at the proximal breakpoint (PB, A) and the distal breakpoint (DB, B) were labeled with arrows. Fig. S2 A schematic diagram of genotyping chromosomal inversions in different peach cultivars. Fig. S3 Schematic diagram for identification of H1 and H2 haplotypes at the S locus based on Illumina HiSeq reads. Fig. S4 The volcano and scatter plots of DEGs. Genes with an adjusted log2 fold change (FC)> 2 and false discovery rate (FDR) < 0.05 were deemed as differentially expressed. Fig S5 PpTRM genes in the peach genome. A, Expression of PpTRM genes in flat‐ and round‐shaped fruits at the S2‐2 stage. Fig S6 Expression levels of PpOFP1 at three different fruit developmental stages derived from a previous RNA‐Seq study (Guo et al., 2018). ‘Zao Huang Pan Tao’ and ‘Zhong Tao Hong Yu’ are flat and round peach cultivars, respectively. Fig. S7 Analysis of RNA in situ hybridization for localization of PpOFP1 mRNA in fruit of ‘124 Pan’ at the S2‐2 stage. Fig. S8 Analysis of interaction between OFPs and TRMs using the yeast two‐hybrid system. Fig. S9 Expression of PpLRR‐RLK and PpCAD1 in fruits at the S2‐2 stage of various peach cultivars. [file PBI-19-192-s002.doc]

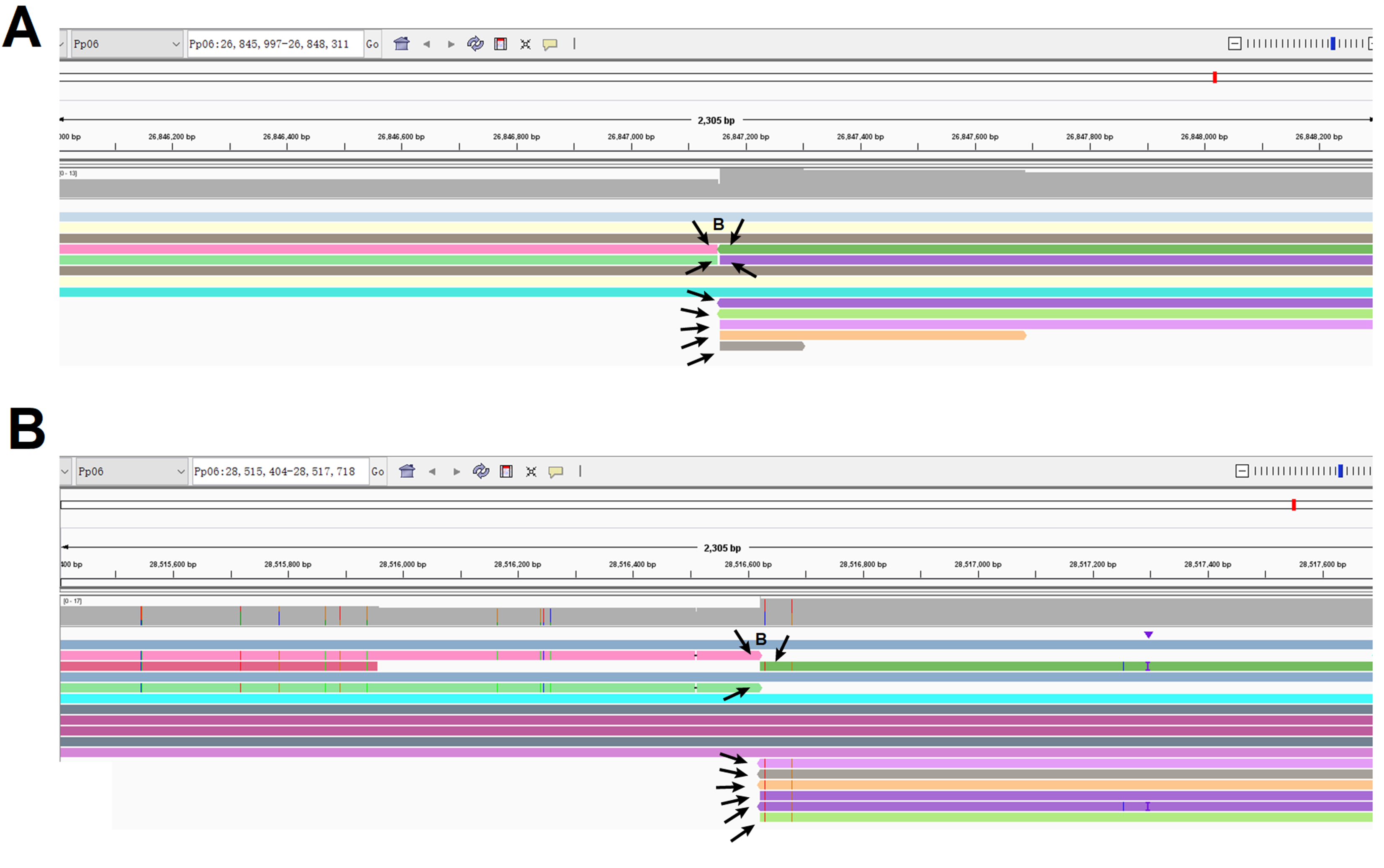


Fig. S1 The PacBio subreads around the breakpoints of the INV. Subreads across the INV were labeled with arrows at the proximal breakpoint (A) and the distal breakpoint (B) regions. The label ‘B’ stands for locations of the breakpoints.


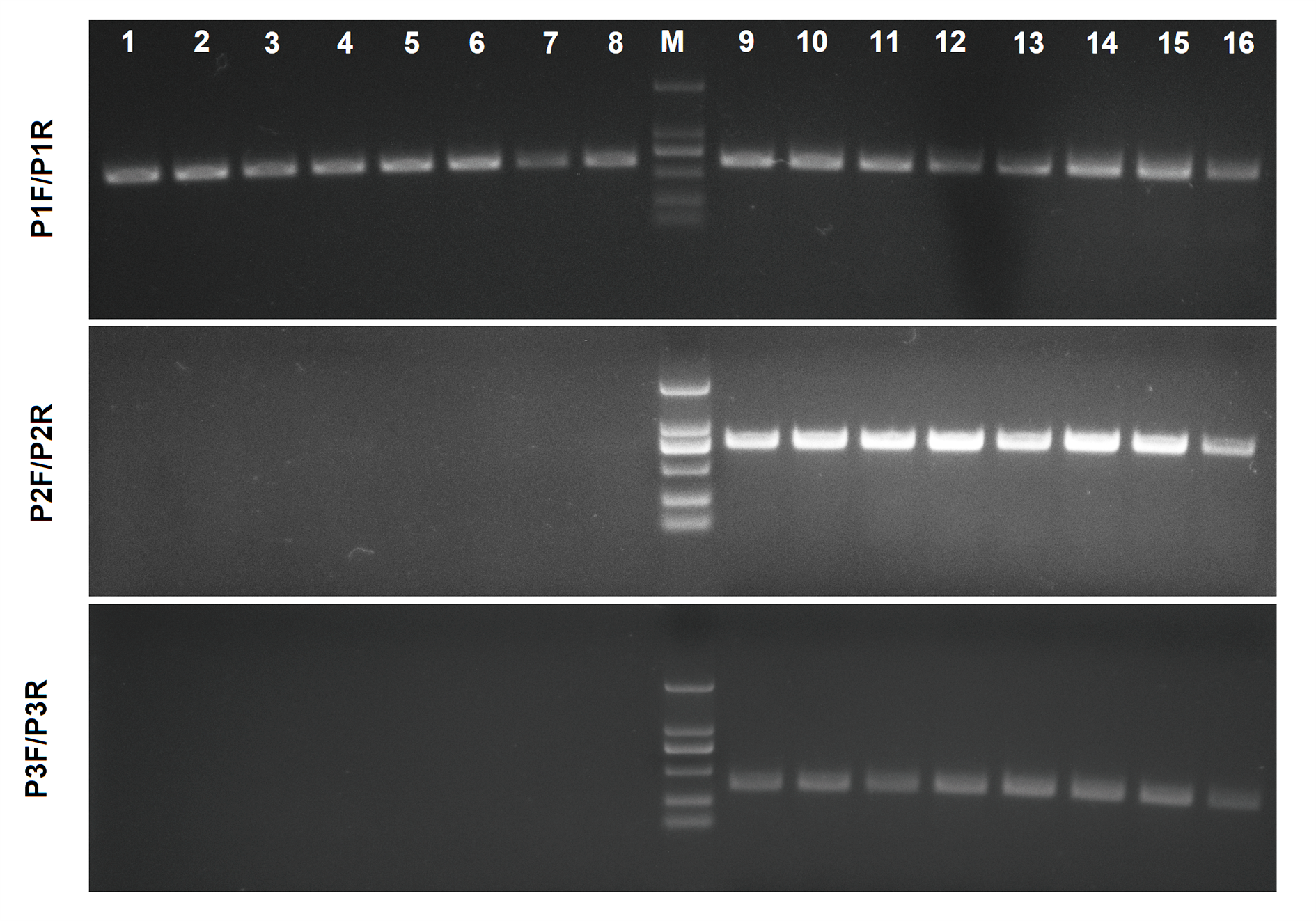


Fig. S2. A schematic diagram of genotyping chromosomal inversions in different peach cultivars. Lanes 1 to 8 correspond to eight round peach cultivars, while lanes 9 to 16 correspond to eight flat peach cultivars. Lanes 1, ‘Wanmi’; 2, ‘Ruiguang 18’; 3, ‘Shenhong’; 4, ‘Hongyingshuang’; 5, ‘Zhongtao 3’; 6, ‘Annongshuimi’; 7,’Zhongguoshahong 1’; 8, ‘Chunxue’; M, DNA ladder; 9, ‘Ruipan 18’; 10, ‘Fangshanpan’; 11, ‘Yuxiapan’; 12, ‘124Pan’, 13, ‘Zaolupan’; 14, ‘Mingyuepan’; 15, ‘Changshengpan’; and 16, ‘Roupan’.


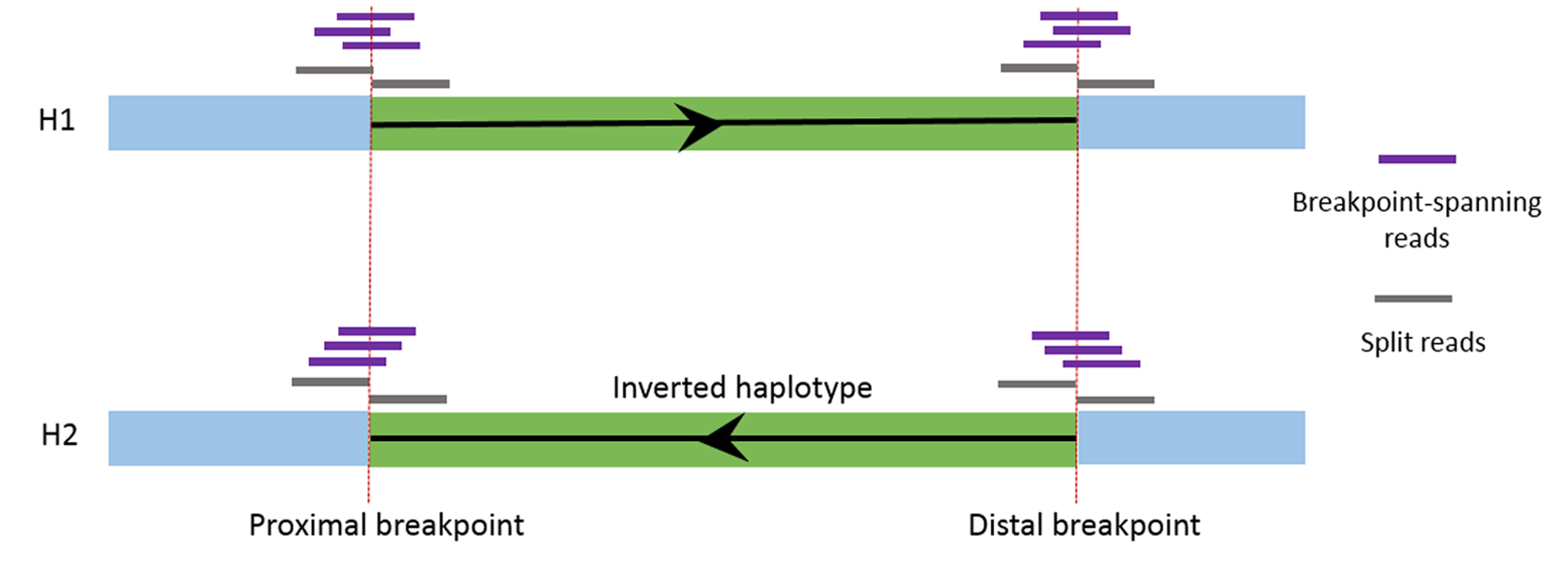


Fig. S3. Schematic diagram for identification of H1 and H2 haplotypes at the S locus based on Illumina HiSeq reads. Breakpoint-spanning reads contain at least 5 bp on each side, while spit reads contain unmatched sequences across the breakpoint.


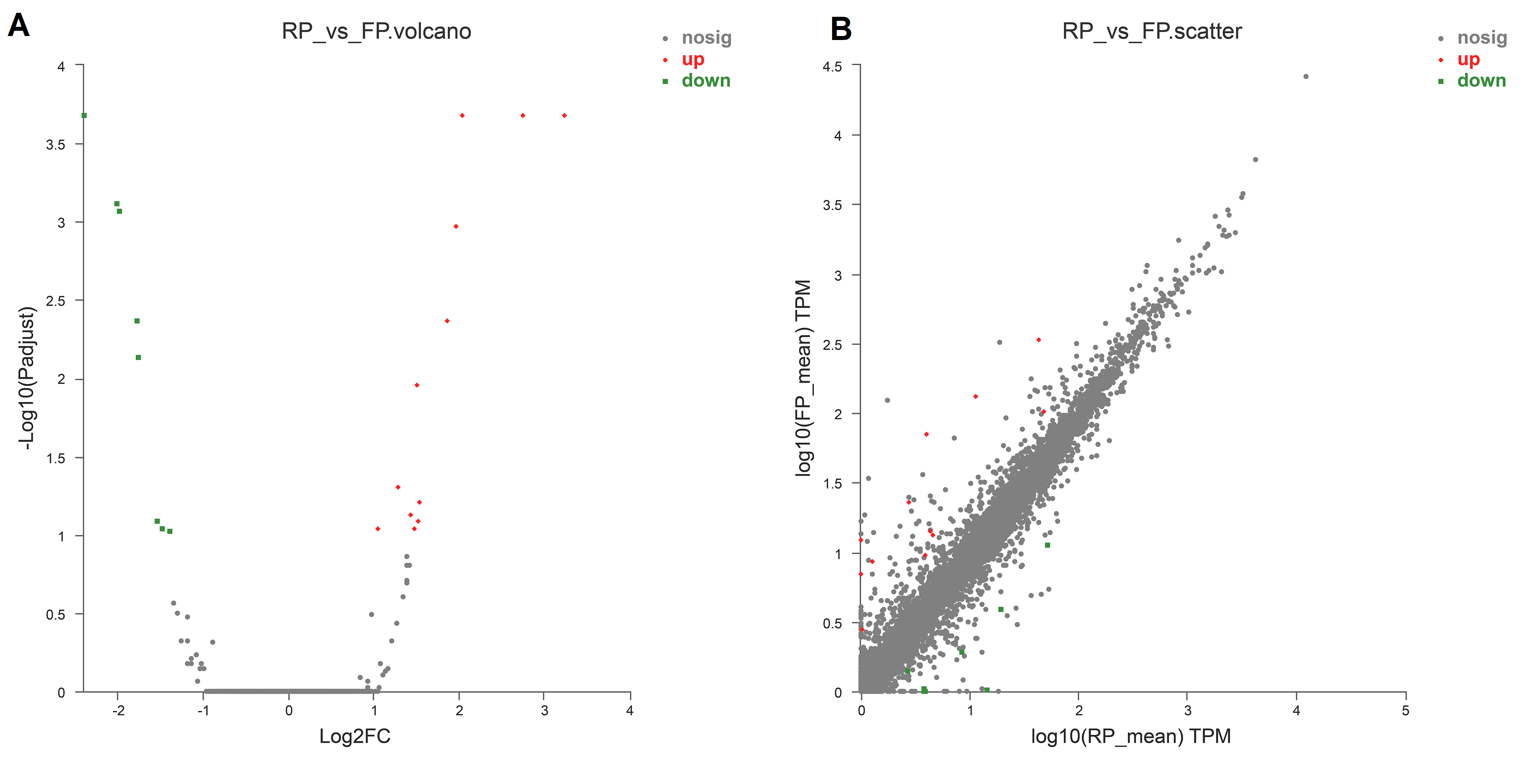


Fig. S4. The volcano and scatter plots of DEGs. Genes with an adjusted log2 fold change (FC) > 2 and false discovery rate (FDR) < 0.05 were deemed as differentially expressed.


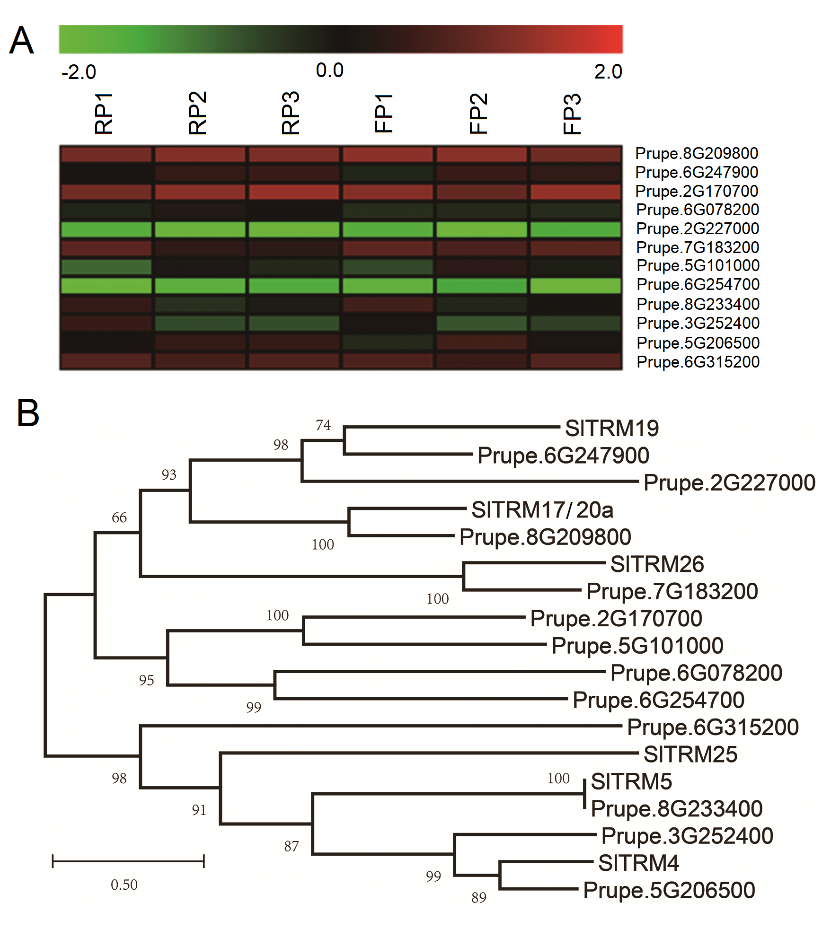


Fig. S5 *PpTRM* genes in the peach genome. A, Expression of *PpTRM* genes in flat- and round-shaped fruits at the S2-2 stage. FP1 to FP3 represent RNA-seq libraries of different flat peach varieties, while RP1 to RP3 represent RNA-seq libraries of different round peach cultivars. B, A phylogenetic tree derived from amino acid sequences of *TRMs* present in both peach and tomato.


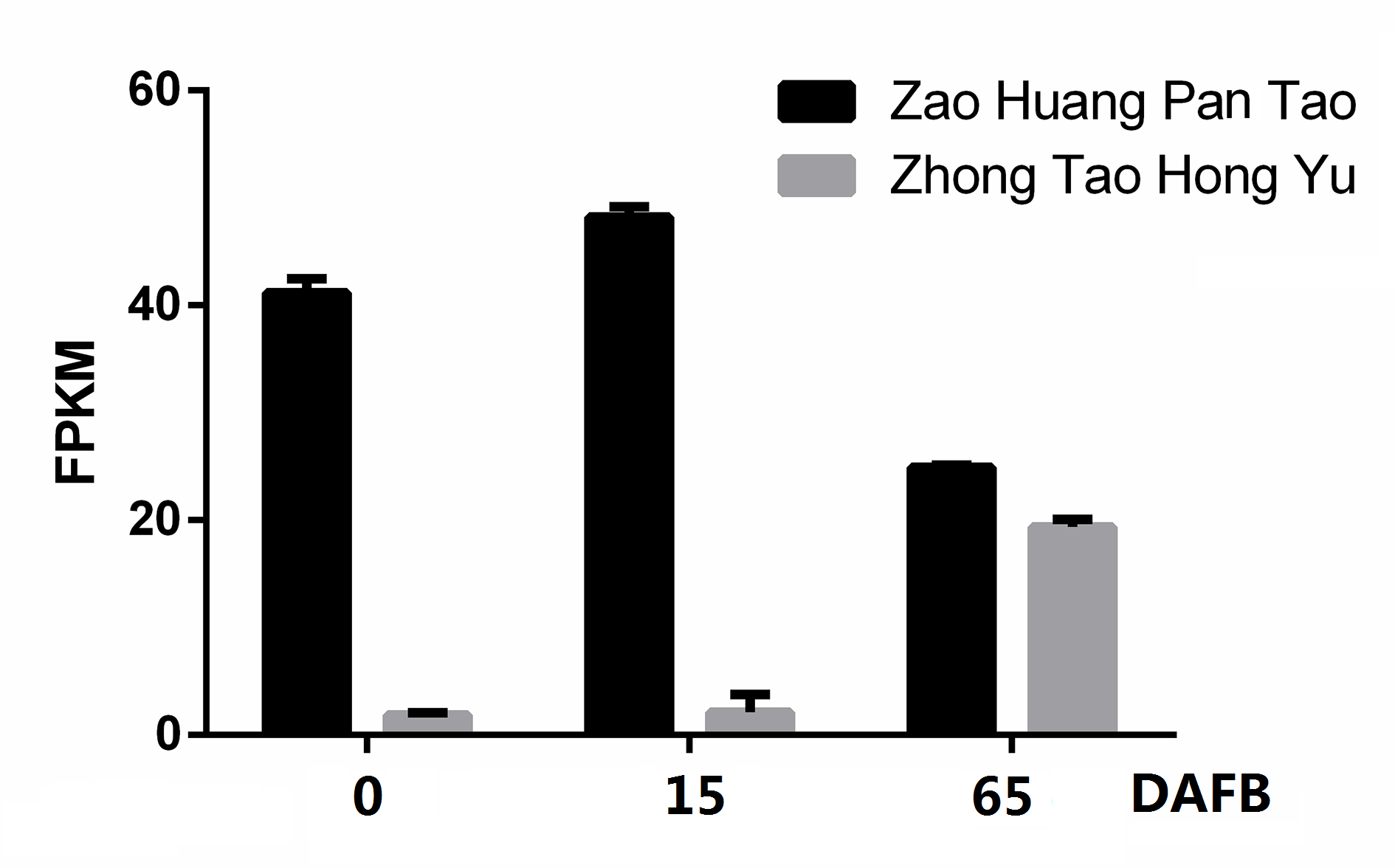


Fig. S6. Expression levels of *PpOFP1* at three different fruit developmental stages derived from a previous RNA-Seq study (Guo *et al*., 2018). ‘Zao Huang Pan Tao’ and ‘Zhong Tao Hong Yu’ are flat and round peach cultivars, respectively.


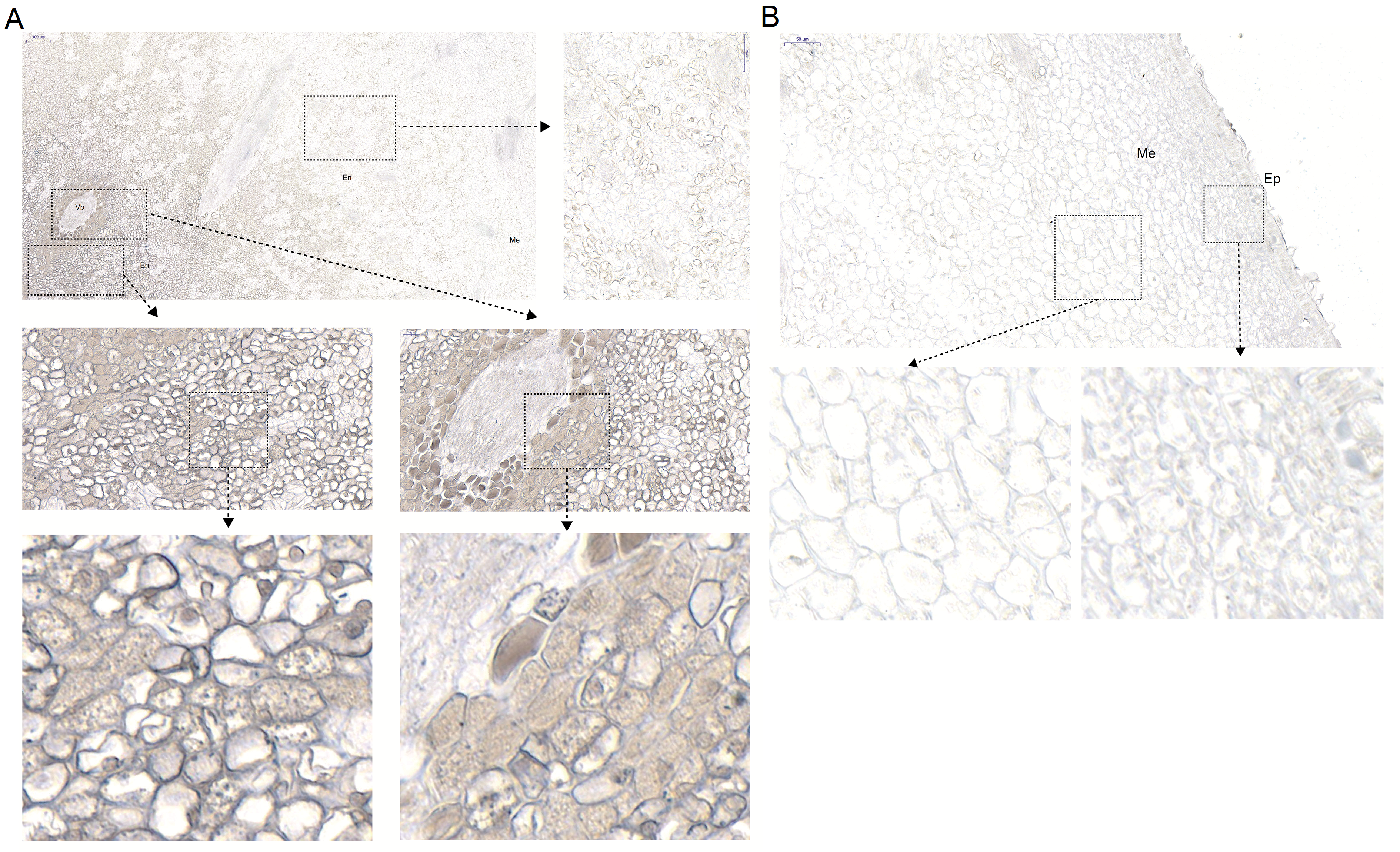


Fig. S7 Analysis of RNA in situ hybridization for localization of *PpOFP1* mRNA in fruit of ‘124 Pan’ at the S2-2 stage. A, Localization of the *PpOFP1* mRNA in endocarp and mesocarp. B, Localization of the *PpOFP1* mRNA in epicarp and mesocarp. Ep, epicarp; Me, mesocarp; En, endocarp; and Vb, vascular bundle.


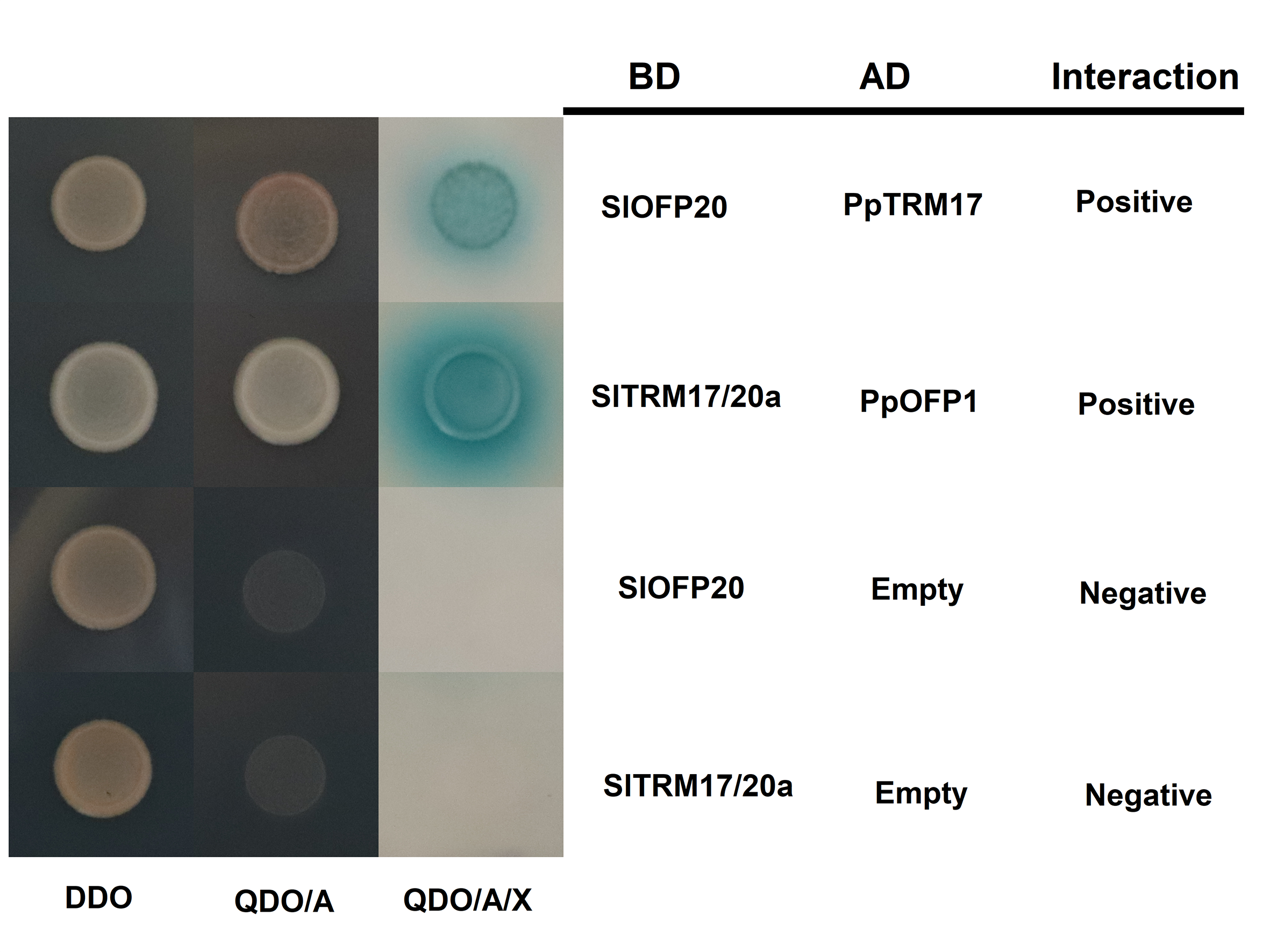


Fig. S8 Analysis of interaction between OFPs and TRMs using the yeast two-hybrid system.


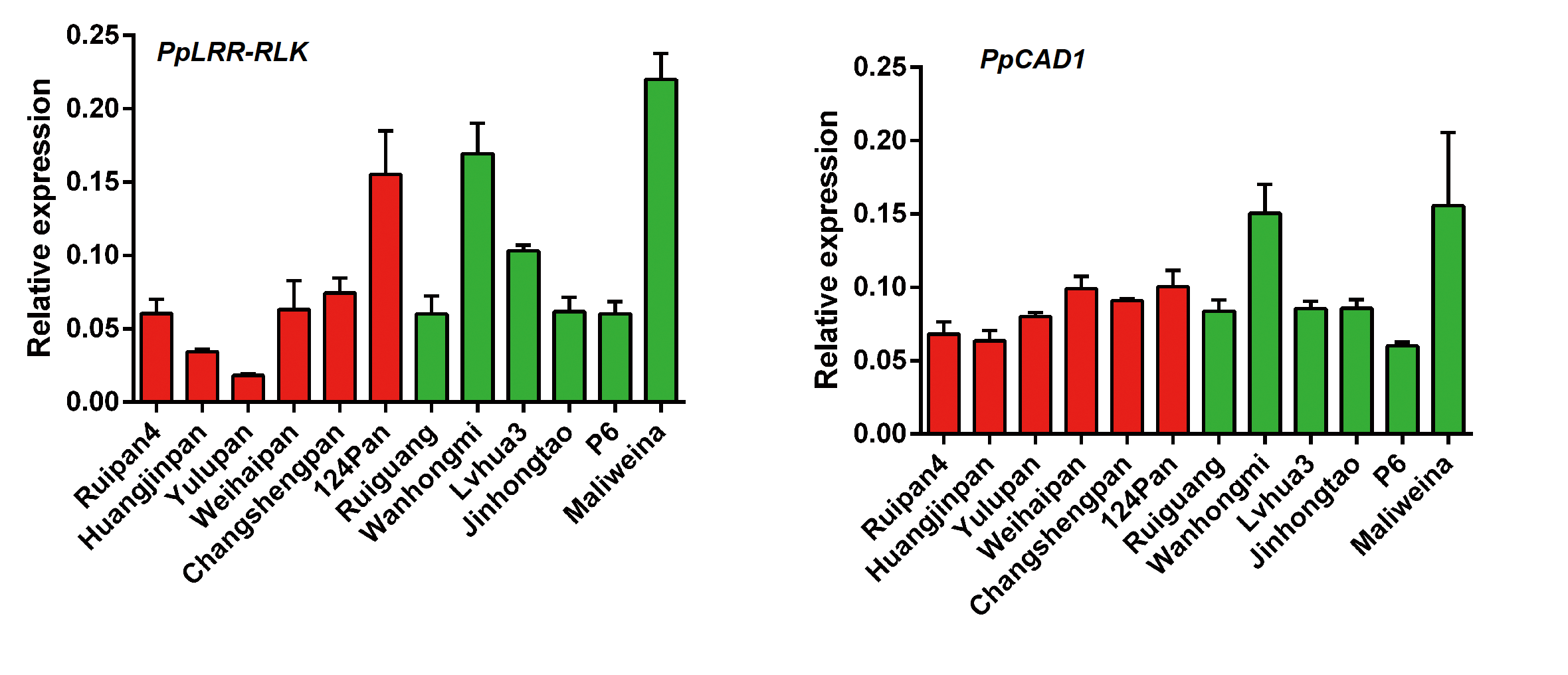


Fig. S9 Expression of *PpLRR-RLK* and *PpCAD1* in fruits at the S2-2 stage of various peach cultivars. Error bars represent SE of three biological replicates. Accessions numbers of *PpLRR-RLK* and *PpCAD1* are *Prupe.6G281100* and *Prupe.6G292200*, respectively.
